# Transcriptomic analysis reveals the key role of inflammatory and immune signaling in the anti-perimenopausal depression effects of Bushen Shugan Huayu decoction

**DOI:** 10.3389/fpsyt.2025.1629900

**Published:** 2025-09-26

**Authors:** Ziqin Feng, Xiaohan Chen, Fengquan Xu, Yicheng Liu, Yu Zheng

**Affiliations:** Guang’anmen Hospital, China Academy of Chinese Medical Sciences, Beijing, China

**Keywords:** Bushen Shugan Huayu decoction, transcriptomic analysis, perimenopausal depression, inflammation, immunity

## Abstract

**Background:**

This study aims to explore the targets and signaling pathways significantly associated with perimenopausal depression through transcriptomics, as well as the potential intervention targets of the BSSGHY decoction.

**Methods:**

Five patients diagnosed with perimenopausal depression in Beijing were treated with the BSSGHY decoction and clinically observed. The severity of depression and associated symptoms was assessed using the HAMD, Kupperman, and PSQI scales before and after treatment, and serum levels of inflammatory factors were quantified using ELISA. Differentially expressed genes in PBMCs were identified through mRNA sequencing and subsequently analyzed using DESeq2 software. Statistical analyses were performed using GraphPad Prism 10.0, and GO and KEGG enrichment analyses were performed based on the hypergeometric distribution algorithm. Key differentially expressed genes were validated via RT-PCR (qPCR) to quantify mRNA expression levels.

**Results:**

Following BSSGHY decoction treatment, patients showed a significant reduction in HAMD scores (p < 0.01), marked symptom relief (p < 0.05), and decreased inflammatory factor levels (p < 0.01). We then conducted RNA-SEQ analysis before and after treatment in patients with perimenopausal depression. Inflammation-related genes such as CXCL8, IL1B, FOSL1, and OSM showed higher expression before treatment, with a downward trend afterward. The analysis of GO and KEGG pathways of differentially expressed genes showed that the involved biological processes and signaling pathways were closely related to inflammation and immunity. qRT-PCR validation showed that FOSL1 and OSM expression decreased after treatment (p < 0.05), while LINC01311 expression increased (p > 0.05).

**Conclusion:**

BSSGHY decoction effectively regulates inflammatory and immune factor signaling, reduces the body’s inflammatory response, and improves perimenopausal depression.

## 1 Introduction

Depression constitutes the primary cause of disability associated with female diseases globally (1). It is estimated that approximately 20% of women will undergo severe depression throughout their lifetimes (2). Menopause represents a natural and inevitable phase in the aging process for women, and the risk of depression among women at this stage escalates significantly. Research has indicated that the risk of developing depressive symptoms or disorders during the perimenopausal period is two to four times higher (3, 4).

Perimenopause typically begins around the age of 47 and is characterized by female reproductive aging and the loss of ovarian hormones, particularly estrogen. Women may present with symptoms associated with menopause, such as hot flashes, night sweats, urogenital symptoms, sleep disturbances, mood changes, and depression (5). The reasons why women in perimenopause are more prone to depression are unclear, which may be related to various neurological, endocrine, genetic, behavioral, and social factors (6). Given that bioenergetic defects and chronic low-grade inflammation are hallmarks of brain aging and menopause, and are considered unified factors causally linking the genetic risk factors of major depressive disorder (MDD) and various neurodegenerative diseases (7), neuroinflammation and immunity are closely implicated in the pathogenesis of perimenopausal depression.

Estrogen is a key regulator of immune and inflammatory processes in women (8). The disruption or loss of estrogen during perimenopause contributes to the development of neuroinflammation during this stage of life. Decreased estrogen levels promote the production of inflammatory cytokines (TNF-α, IL-1β), exacerbating the neuroinflammation already associated with aging (9). Furthermore, inflammasomes originating in the female reproductive system may further contribute to neuroinflammation (10). Multiple studies suggest that the modulation of neuroinflammation by estrogen is mediated through signaling pathways such as ERα/SIRT1/NF-κB and AMPK/NF-κB (11, 12). A deeper understanding of these molecular mechanisms underlying estrogen’s influence on depression may contribute to the development of novel therapeutic strategies for depression and provide potential targets for treating perimenopausal depression (13).

Based on its clinical manifestations, PMD (perimenopausal depression) can be classified under the categories of ‘Yu Zheng’ (depressive syndrome), ‘Zang Zao’ (restless organ syndrome), and ‘symptoms before and after menopause’ within the framework of traditional Chinese medicine (TCM). Upon exploring TCM syndromes in perimenopausal depression patients, kidney deficiency with liver qi stagnation and blood stasis were found to be common syndromes (14). These findings suggest that kidney deficiency, liver qi stagnation, and blood stasis constitute the fundamental pathogenesis of PMD (15), with ‘kidney deficiency’ as the root cause, ‘liver qi stagnation’ as the external manifestation, and ‘blood stasis’ as an important pathological condition. Based on this theory, the Bushen Shugan Huayu decoction(BSSGHY decoction) was formulated to treat perimenopausal depression. The main ingredients of BSSGHY decoction include Curculiginis Rhizoma(Xianmao, Chinese), Epimedii Folium (Yinyanghuo, Chinese), Ligustri Lucidi Fructus(Nüzhenzi, Chinese), Ecliptae Herba(Mohanlian, Chinese), Bupleuri Radix(Chaihu, Chinese), Aurantii Fructus(Zhiqiao, Chinese), Chuanxiong Rhizoma (Chuanxiong, Chinese), and Pheretima(Dilong, Chinese), with details of its dosage and administration provided in **Supplementary Table S1**. Clinically, BSSGHY decoction has demonstrated significant efficacy in treating perimenopausal depression (PMD), alleviating depressive symptoms, improving perimenopausal manifestations, and significantly reducing follicle-stimulating hormone (FSH) and luteinizing hormone (LH) levels (16–18). Although its precise mechanism of action remains unclear, BSSGHY decoction may exert antidepressant effects in postmenopausal women by modulating central monoamine neurotransmitter levels.

The emergence of next-generation sequencing technologies has significantly advanced our understanding of disease pathogenesis and drug mechanisms, thereby offering new avenues to mitigate the ongoing risks associated with current interventions. In particular, transcriptomics allows for comprehensive exploration of gene expression in specific tissues and at specific times, ushering in a transformative era in medical research. A distinguishing characteristic of TCM intervention in diseases is its multi-target, multi-step, and multi-phase approach. Utilizing next-generation sequencing can help accurately pinpoint the key targets and phases of TCM interventions. To this end, blood samples were collected from perimenopausal depression patients treated with BSSGHY decoction, and transcriptomic sequencing analysis was conducted based on treatment outcomes. The sequencing results will be applied to further investigate the pathogenesis of PMD, identify potential therapeutic targets, and elucidate pathways of BSSGHY decoction in treatment and intervention.

## 2 Materials and methods

### 2.1 Subjects

The study protocol was approved by the Ethical Committee, Guang’an men Hospital, China Academy of Chinese Medical Sciences(2024-130-KY-01), and written informed consent was obtained. This project has been registered on the International Traditional Medicine Clinical Trial Registry, with the registration number ITMCTR2024000385.

### 2.2 Participants

We recruited patients diagnosed with perimenopausal depression at Guang’anmen Hospital between July and September 2024. Participants met the following criteria:①voluntary participation with signed informed consent;②women aged 45–65 years in the perimenopausal stage;③diagnosis of depression according to the Chinese Classification of Mental Disorders (3rd Edition) with a Hamilton Depression Rating Scale (HAMD) score of 8–34;④meets the diagnostic criteria for pre- and post-menopausal syndromes of kidney deficiency, liver depression, and blood stasis, as described in Traditional Chinese Gynecology;⑤treatment with the BSSGHY decoction;⑥no use of estrogen-based medications or discontinuation of such medications for several months; and⑦no use of psychiatric medications or discontinuation for at least one month.

Patients were excluded if they met any of the following criteria:①presence of other serious organic diseases or mental disorders;②life-threatening cardiovascular or cerebrovascular diseases;③history of alcohol or drug abuse or dependence within the past 3 months;④high risk of suicide as determined by clinical judgment;⑤history of total hysterectomy and oophorectomy; or⑥participation in another drug clinical trial within one month prior to enrollment.

### 2.3 Drug formulation and administration

The BSSGHY decoction, in granule form, was provided by the Department of Pharmacy at Guang’an men Hospital. The specific dosages are detailed in **Supplementary Table S1**. The BSSGHY decoction was administered orally at a dose of 200 mL twice daily for one month.

### 2.4 Clinical data collection

Baseline information, including gender, height, weight, and marital status, was collected prior to treatment. Depressive status was assessed using the 24-item Hamilton Depression Rating Scale (HAMD-24) at baseline and one month after treatment, where a score < 8 indicates no depression, 8–19 indicates mild depression, 20–34 indicates moderate depression, and a score ≥ 35 indicates severe depression. Sleep quality was evaluated using the Pittsburgh Sleep Quality Index (PSQI), which consists of 18 items with a total score range of 0-21; the higher the score, the poorer the sleep quality. The modified Kupperman index was used to assess patient symptoms, including fatigue, paresthesia, hot flashes, sweating, insomnia, palpitations, and 13 other items, where lower scores indicate milder symptoms. All assessments were conducted by independent medical professionals. Serum concentrations of IL-6 and TNF-α were measured by ELISA.

### 2.5 RNA extraction and analysis

Peripheral blood mononuclear cells (PBMCs) were isolated from the collected whole blood using the density gradient centrifugation method. Total RNA was extracted from PBMCs using TRIzol reagent according to the manufacturer’s instructions. The purity and quantification of RNA were assessed using a NanoDrop 2000 spectrophotometer, and the RNA integrity was evaluated using an Agilent 2100 Bioanalyzer. Transcriptome libraries were constructed using the VAHTS Universal V5 RNA-seq Library Prep Kit according to the manufacturer’s instructions. The libraries were sequenced on the Illumina Novaseq 6000 sequencing platform, generating 150 bp paired-end reads. Approximately 48M raw reads were obtained for each sample.

The fastp software was used to process the raw reads in fastq format, and clean reads were obtained after removing low-quality reads for subsequent data analysis. The HISAT2 software was used for reference genome alignment and gene expression quantification (FPKM), and the read counts (counts) for each gene were obtained using HTSeq-count. PCA analysis and plotting were performed on the gene (counts) using R (v 3.2.0) to evaluate the biological replicates of the samples.

### 2.6 Quantitative RT-PCR

Reverse transcription was performed using the TransScript All-in-one First-Strand cDNA Synthesis SuperMIX for qPCR kit according to the manufacturer’s instructions. Reactions were carried out on a LightCycler^®^ 480 II real-time PCR instrument using the PerfectStart™ Green qPCR SuperMix kit. The expression levels were calculated using the 2-ΔΔCt method.

### 2.7 Data analysis

Differentially expressed gene (DEG) analysis was performed using the DESeq2 software, where genes meeting the thresholds of p-value < 0.05 and |log2FC| > 0.26 were defined as differentially expressed genes (DEGs). GO and KEGG enrichment analyses of DEGs were conducted based on the hypergeometric distribution algorithm to identify significantly enriched functional terms.

Data are presented as mean ± SEM. Clinical analyses were performed using SPSS 26.0, and results were visualized with GraphPad Prism 10.0. Between-group comparisons were conducted using t-tests, with p < 0.05 considered statistically significant.

## 3 Results

### 3.1 Clinical data

**Figure 1:**
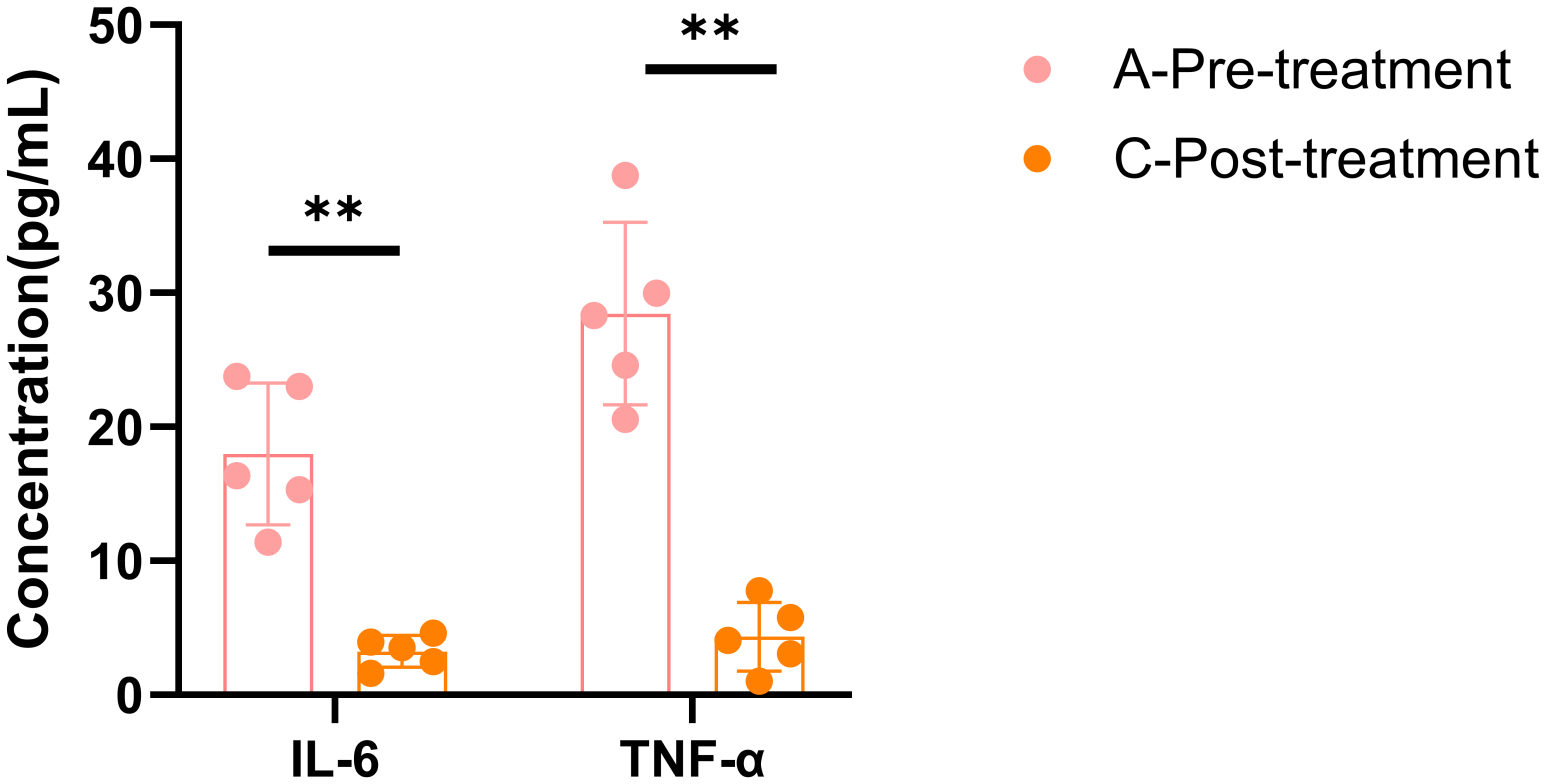
ELISA results for IL-6 and TNF-α. Data are shown as mean ± SD, **p < 0.01 vs. post-treatment.

A total of 5 female patients, aged between 45 and 51, were enrolled in this study. Before treatment, their HAMD scores ranged from 9 to 27. After treatment, the HAMD scores significantly decreased (p < 0.01), and symptoms were alleviated to some extent (p < 0.05) (**Table 1**). This is consistent with previous results, suggesting that the BSSGHY decoction is quite effective. It not only improves depressive symptoms in patients but also alleviates physical symptoms. Additionally, we observed that the patients’ accompanying symptoms mainly included fatigue, insomnia, and pain (such as headaches, joint pain, and muscle pain). Studies suggest that the occurrence of these symptoms may be closely related to inflammation (19–22). Consistent with these findings, IL-6 and TNF-α levels were elevated in patients before treatment compared with normal levels, but were significantly reduced after treatment (p < 0.01) (**Figure 1**).

**Table 1:** Basic patient information and therapeutic effect index.

Basic information		Treatment phase	Therapeutic effect index		
Age	BMI		HAMD score	Kupperman score	PSQI score
48.60 ± 2.88	21.62 ± 1.84	Pre-treatment	16.80 ± 7.56	16.40 ± 7.02	11.20 ± 5.26
		Post-treatment	8.40 ± 4.28	9.60 ± 4.62	5.00 ± 3.00

### 3.2 Comparison of differentially expressed genes before and after treatment with BSSGHY decoction

**Figure 2:**
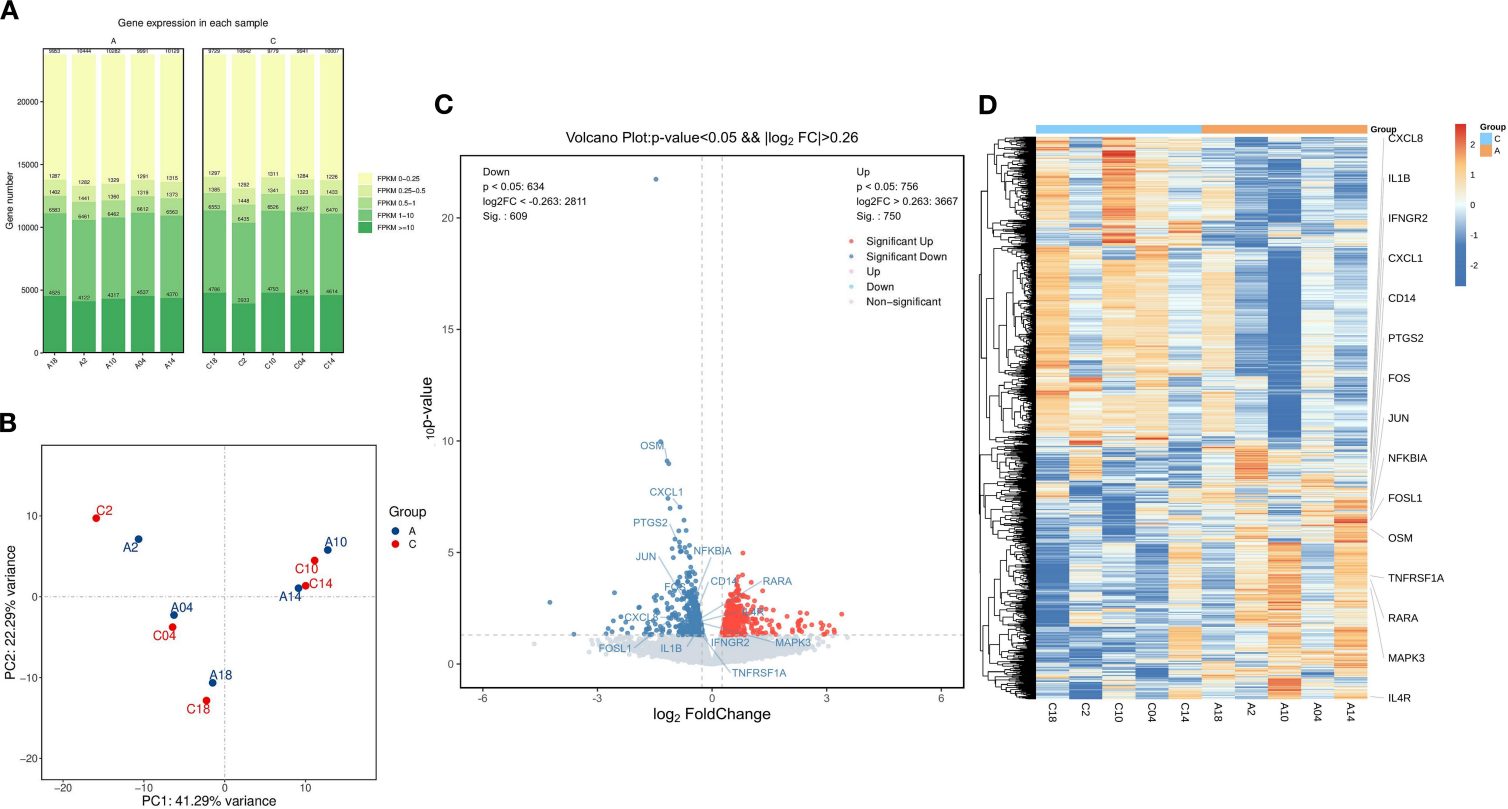
Comparison of Differentially Expressed Genes Before and After Treatment with the BSSGHY decoction. **(A)** Distribution plot of expression levels (FPKM) for each sample before and after treatment. **(B)** Principal component analysis (PCA) plot before and after treatment, with A representing pre-treatment and C representing post-treatment. **(C)** Volcano plot of differentially expressed genes, with red indicating significant upregulation and blue indicating significant downregulation. **(D)** Heatmap of hierarchical clustering of differentially expressed genes, with red representing relatively highly expressed protein-coding genes and blue representing relatively lowly expressed protein-coding genes.

To investigate the gene expression profiles before and after treatment with the BSSGHY decoction, we performed RNA-Seq analysis on peri-menopausal depression patients pre- and post-treatment. The number of genes detected before and after treatment in peri-menopausal depression patients was approximately the same, fluctuating around 19,000, with no issues regarding sample quality, as shown in **Figure 2A**. The samples were tested in pairs, and the principal component analysis (PCA) plot indicated differences between pre- and post-treatment samples, suggesting that the treatment effectively alleviated patient symptoms. However, due to the short treatment period, the full effect of the herbal medicine may not have been realized, resulting in relatively small differences between the pre- and post-treatment groups, as shown in **Figure 2B**.

Using thresholds of |log2FC| > 0.26 and P-value < 0.05, we identified 1,359 differentially expressed genes (DEGs), of which 750 genes were upregulated and 609 were downregulated, as shown in **Figure 2C** and **Supplementary Table S2**. A heatmap of the hierarchical clustering of DEGs revealed that, although biological variability existed, there were distinct differences in clustering between the pre- and post-treatment groups. Additionally, inflammation-related genes such as CXCL8, IL1B, FOSL1, and OSM were highly expressed before treatment and showed a downward trend after treatment, as shown in **Figure 2D**.

### 3.3 GO function and KEGG pathway analysis

**Figure 3:**
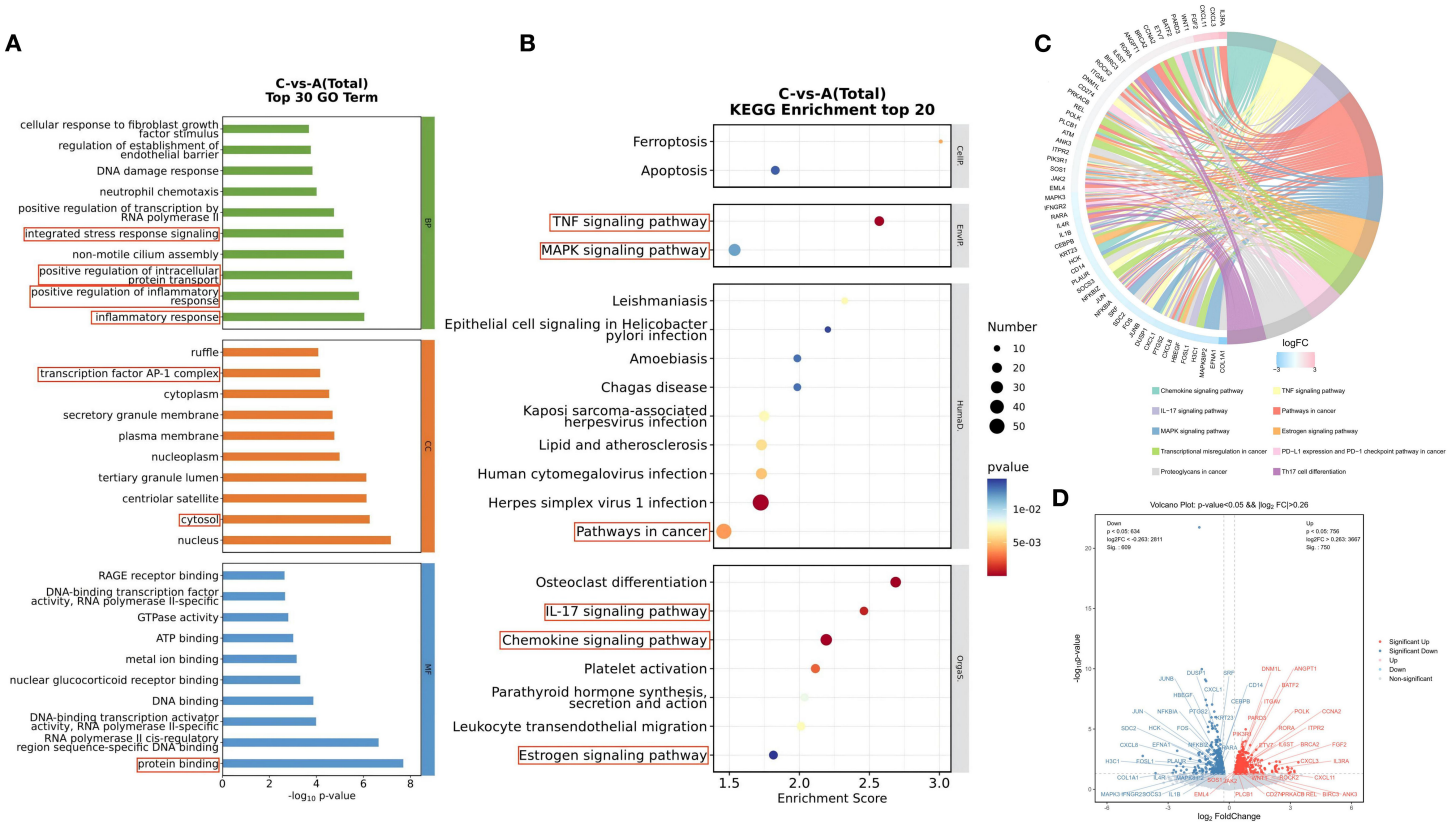
GO Function and KEGG Pathway Analysis of Differentially Expressed Genes Before and After Treatment with the BSSGHY decoction. **(A)** Top 30 GO enrichment analysis of differentially expressed genes, with circles highlighting inflammation-related biological functions. **(B)** Top 20 KEGG enrichment analysis of differentially expressed genes, with circles highlighting inflammation-related signaling pathways. **(C)** KEGG enrichment analysis chord diagram, showing pathways with p<0.05. The left side shows the top 10 genes with the largest |logFC| in each pathway, while the right side reflects the composition of each pathway. The lines in the middle indicate the relationship between pathways and genes. The outer heatmap represents the logFC values of the corresponding genes. **(D)** Volcano plot of differentially expressed genes in the pathways shown in the chord diagram, with red indicating significant upregulation and blue indicating significant downregulation.

GO and KEGG enrichment analyses were used to evaluate and predict the potential functions of the identified differentially expressed genes (DEGs). All GO and KEGG pathways meeting the significance criterion of p < 0.05 are presented in **Supplementary Tables S3, S4**. The top 30 GO terms are shown in **Figure 3A**. The top three ranked cellular component (CC) terms were nucleus, cytosol, and centriolar satellite. The top three ranked biological process (BP) terms were inflammatory response, positive regulation of inflammatory response, and positive regulation of intracellular protein transport. For molecular function (MF), the top three ranked terms were protein binding, RNA polymerase II cis-regulatory region sequence-specific DNA binding, and DNA-binding transcription activator activity, RNA polymerase II-specific.

Among the top 30 GO terms, many were related to inflammatory and immune processes, such as inflammatory response, positive regulation of inflammatory response, positive regulation of intracellular protein transport, integrated stress response signaling, transcription factor AP-1 complex, cytosol, and protein binding. These findings suggest that the biological processes associated with the differentially expressed genes before and after treatment with the BSSGHY decoction are closely related to inflammation and immunity.

KEGG pathway analysis showed the top 20 signaling pathways involving DEGs, as illustrated in **Figure 3B**. These pathways primarily included ferroptosis, apoptosis, estrogen signaling pathway, chemokine signaling pathway, virus infection-related pathways, TNF, MAPK, and IL-17 signaling pathways. Pathways related to inflammation and immune responses (p<0.05) and the DEGs enriched in these pathways are shown in **Figure 3C**. In addition to the top 20 pathways, other pathways included Th17 cell differentiation, transcriptional misregulation in cancer, proteoglycans in cancer, and PD-L1 expression and PD-1 checkpoint pathway in cancer. We used a volcano plot to provide a detailed visualization of gene expression changes in key pathways (**Figure 3D**). Among the DEGs enriched in these pathways, upregulated genes included IL3RA, CXCL3, CXCL11, and FGF2, while downregulated genes included COLQA1, EFNA1, MAPK8IP2, and FOSL1.

**Figure 4:**
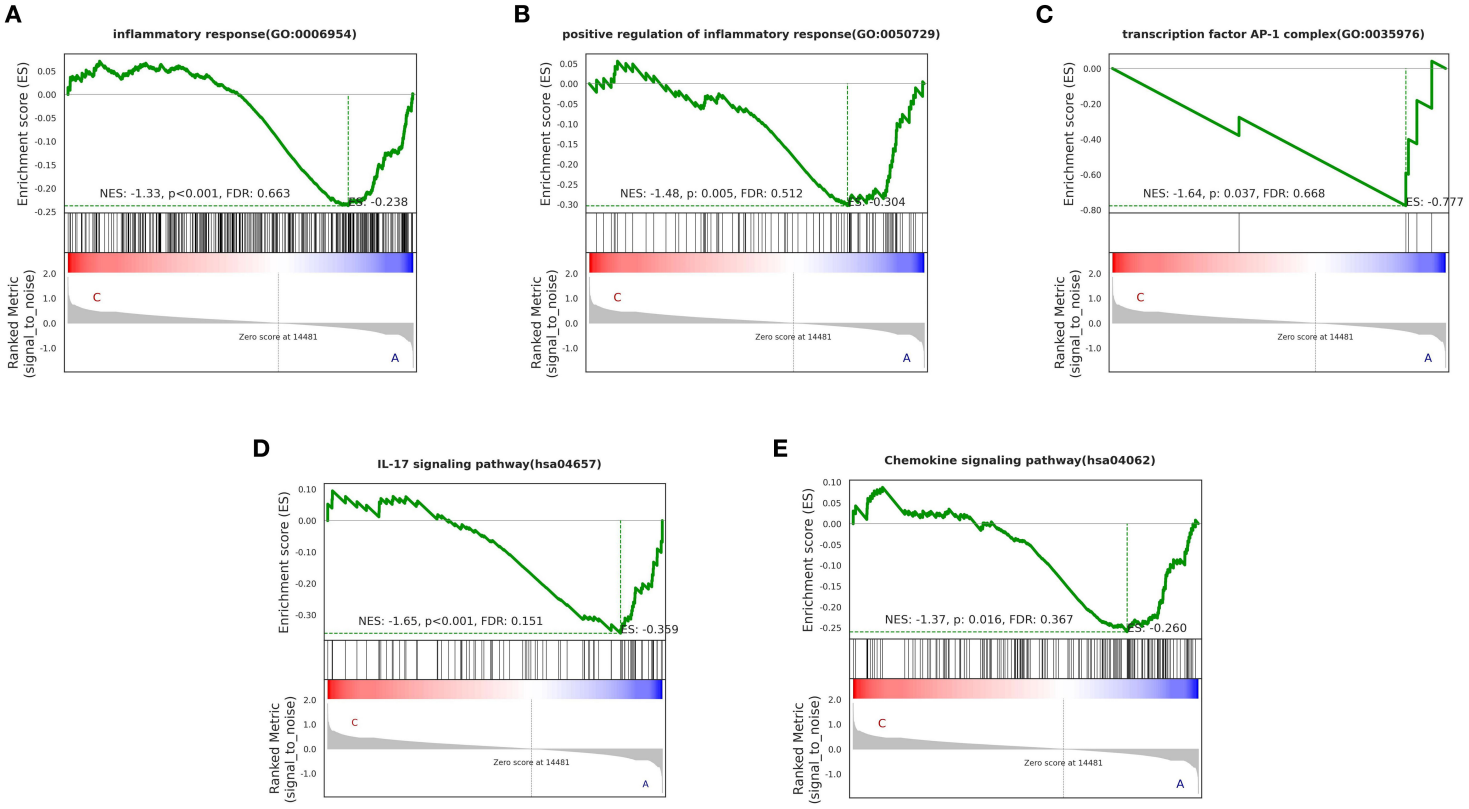
GO and KEGG enrichment results of GSEA for key pathways (p < 0.05). Specific corresponding pathways: **(A)** inflammatory response, **(B)** positive regulation of inflammatory response, **(C)** transcription factor AP-1 complex, **(D)** IL-17 signaling pathway, **(E)** Chemokine signaling pathway. The green line represents the ES (enrichment score) distribution of all genes, with the curve’s peak on the Y-axis indicating the maximum absolute value of the enrichment score for the gene set. If ES>0, core genes are located to the left of the peak, and if ES<0, core genes are located to the right of the peak.

We observed that certain “pro-inflammatory factors” were upregulated after treatment, while “anti-inflammatory factors” were downregulated after treatment. This may be related to local concentrations of factors, different stages of the disease, and interactions with other cytokines (23). However, overall, the BSSGHY decoction reduced the body’s inflammatory response. We performed GSEA on the GO and KEGG signaling pathways of interest. The results showed that inflammation- and immunity-related pathways were suppressed after treatment, consistent with previous findings and indicating that inflammation was alleviated (**Figure 4**). The GO and KEGG enrichment results for key pathways associated with inflammation and immunity, along with the corresponding GSEA results for these pathways, are provided in **Supplementary Tables S5, S6**.

### 3.4 qRT-PCR validation

We used real-time qRT-PCR to validate the expression of three genes. FOSL1 is one of the differentially expressed genes in the IL-17 signaling pathway and is also a component of the AP-1 transcription factor. Additionally, OSM belongs to the IL-6 cytokine family. Although its pathway was not enriched, it is closely related to neuroinflammation and blood-brain barrier damage. Based on these findings, we selected FOSL1 and OSM for qRT-PCR validation.

**Figure 5:**
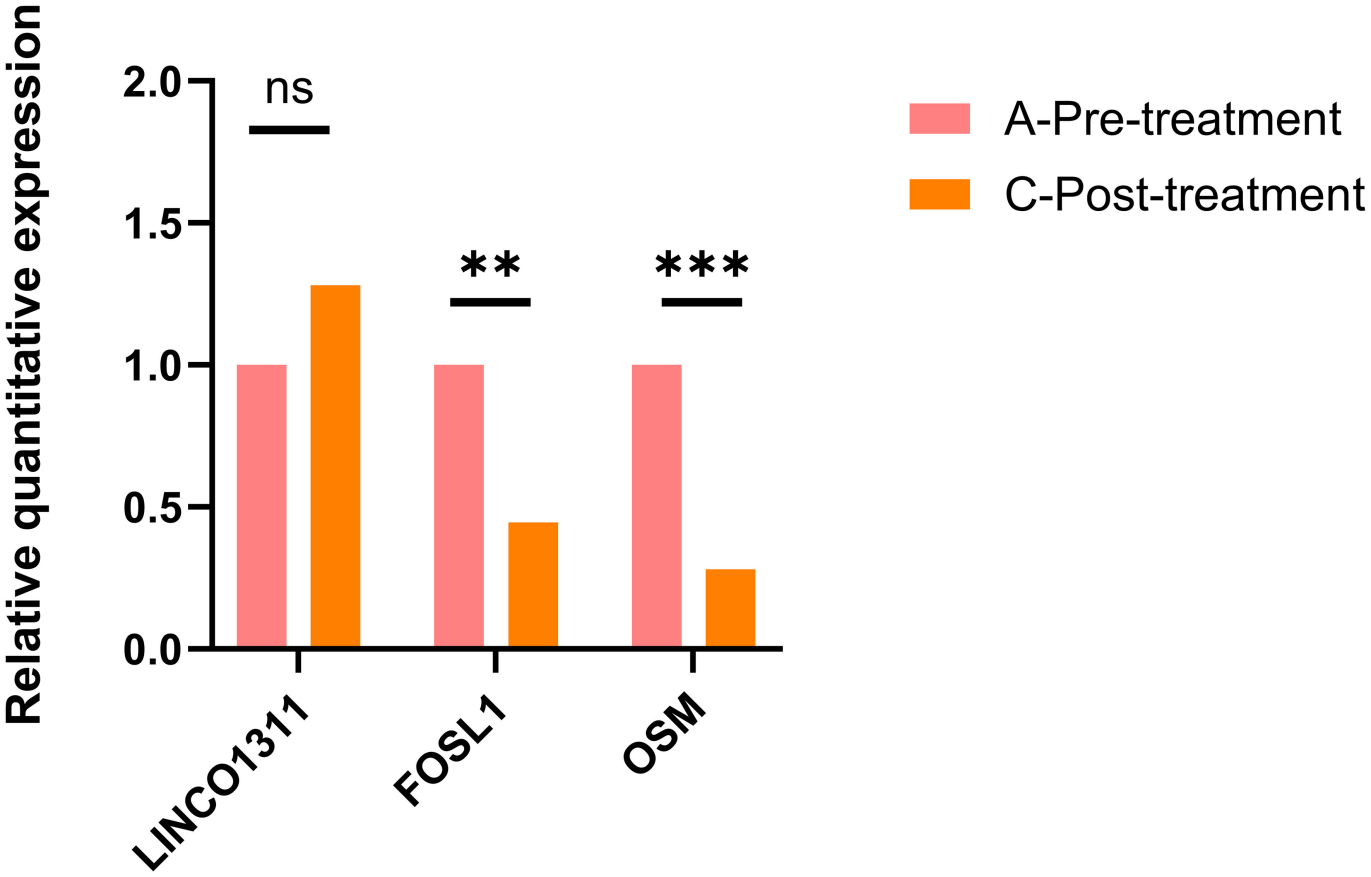
The qRT-PCR validation results for FOSL1, OSM, and LINC01311 showed that LINC01311 had p > 0.05 before and after treatment, while the other two genes had p < 0.05. **p < 0.01 vs. post-treatment; ***p < 0.001 vs. post-treatment; ns, p > 0.05 vs. post-treatment.

Furthermore, we found a significant number of long non-coding RNAs (lncRNAs) among the DEGs. Recent studies have shown that LINC01311 has a protective effect against Alzheimer’s disease-like damage, so we also validated the expression of this gene using qRT-PCR. The results showed that, compared to pre-treatment levels, the expression of FOSL1 and OSM decreased, while the expression of LINC01311 increased, as shown in **Figure 5**.

## 4 Discussion

The potential neuroendocrine mechanisms by which complex hormonal changes during menopause lead to depressive symptoms remain to be studied. However, evidence from current literature suggests that a state of low-grade chronic inflammation during the perimenopausal period may represent a contributing mechanism (24, 25). During the perimenopausal period, women experience a decline in ovarian function and aging, leading to fluctuations and decreases in estrogen levels, activation of immune cells, and the formation of a pro-inflammatory cytokine environment (7), which is one of the reasons why the immune system’s reactivity is affected in perimenopausal women. BSSGHY decoction has been shown to significantly alleviate clinical symptoms such as depressed mood in perimenopausal depression patients, demonstrating notable clinical efficacy. Therefore, we reasonably speculate that neuroinflammation caused by estrogen deficiency may be one of the important mechanisms leading to the occurrence of perimenopausal depression and is also an important mechanism by which BSSGHY decoction treats PMD. Here, we first compared the transcriptome profiles of perimenopausal depression patients before and after treatment with BSSGHY decoction, followed by GO and KEGG enrichment analyses, which showed that the DEGs regulated by BSSGHY decoction are highly related to immune function and inflammation. Meanwhile, patients’ inflammatory factor levels were elevated before treatment and decreased significantly afterward.

The data from this study indicate that, alongside improvements in clinical symptoms and inflammatory factor levels, treatment with BSSGHY decoction also led to significant changes in gene expression. Through GO and KEGG enrichment analyses, we evaluated and predicted the potential functions of the identified DEGs to be related to inflammatory response, regulation of inflammatory response, intracellular protein transport, and integration of stress response signals. Among these, Estrogen signaling pathway, MAPK signaling pathway, Pathways in cancer, PD-L1 expression and PD-1 checkpoint pathway in cancer, TNF signaling pathway, IL-17 signaling pathway, and Th17 cell differentiation, which can be regulated by estrogen, play important roles in immune response, inflammation, cell proliferation, and cancer. Furthermore, DEGs after treatment with BSSGHY decoction, such as FOS, JUN, PIK3R1, RARA, and MAPK3, are directly or indirectly related to the regulation of the estrogen signaling pathway, indicating that the potential pathways of BSSGHY decoction in perimenopausal depression may involve modulation of estrogen-related pathways.

It is well known that the transcription of inflammatory cytokines is regulated by several key transcription factors, including nuclear factor kB (NF-kB), activator protein-1 (AP-1), IL-6, nuclear factors of activated T cells (NFATs), and signal transducers and activators of transcription (STATs) (26). IL-6 is a signaling product of the NF-κB pathway, and after brain injury, the expression of NF-κB is upregulated in neurons, astrocytes, and microglia, thereby inducing increased secretion of inflammatory factors such as IL-6 and triggering local brain inflammation (27). Inhibition of NF-κB, the principal transcription factor regulating pro-inflammatory mediators, is widely regarded as an effective strategy to reduce neuroinflammation by inhibiting the activation of NF-kB (26). We found that after treatment with BSSGHY decoction, the activation of the NF-kB signaling pathway decreased, as evidenced by genes such as CXCL8, PTGS2, NFKBIA, CD14, CXCL1, and TNFRSF1A. These data suggest that the potential pathways of BSSGHY decoction in treating perimenopausal depression are closely related to reducing neuroinflammation.

### 4.1 Estrogen deficiency affects immune function and induces inflammatory responses

Previous studies have shown that estrogen deficiency can lead to changes in the body’s immune function, resulting in exacerbated immune responses mediated by various cytokines (8). Postmenopausal women without other inflammatory diseases have higher levels of IL-1, IL-6, and TNF-α than premenopausal women (28). Numerous studies have confirmed that IL-6 is an important participant in the inflammatory cascade reaction and one of the most multifunctional and widespread inflammatory cytokines. It not only contributes to inflammatory diseases but also has important functions in the nervous system. IL-6 can regulate the development, differentiation, and survival of neurons, and maintain the growth and normal function of the nervous system. Excessive IL-6 can damage nerve cells through its inflammatory action (29). Targeted blockade of IL-6 and its signaling pathways is recognized as an effective strategy for the treatment of various diseases (30). In the present study, we found that among the signaling pathways involved in BSSGHY decoction, Cytokine-cytokine receptor interaction, Viral protein interaction with cytokine and cytokine receptor, Signaling pathways regulating pluripotency of stem cells, JAK-STAT signaling pathway, Th17 cell differentiation, Kaposi sarcoma-associated herpesvirus infection, Coronavirus disease 2019 (COVID-19), Pathways in cancer, and Viral carcinogenesis are all related to IL-6 regulation. Meanwhile, patients showed differences in IL-6 levels before and after treatment. This suggests that perimenopausal depression is mediated by a neuroinflammatory mechanism, and estrogen plays a crucial role in perimenopausal depression through inflammatory and immune factor signaling. BSSGHY decoction may modulate estrogen-related pathways and downregulate IL-6 levels, thereby suppressing inflammation and improving perimenopausal depression.

Subsequently, we used real-time qRT-PCR to validate the expression of two NF-kB and IL-6-related pathway DEGs, and the results showed that compared to pre-treatment, the expression of FOSL1 and OSM was decreased.

### 4.2 Upregulation of FOSL1 mediates an increase in pro-inflammatory factors, leading to chronic inflammatory symptoms

FOSL1 (Fos-like antigen 1) is a member of the Fos family. FOSL1 often binds with Jun proteins to form active AP-1 complexes. Activator protein-1 (AP-1) can regulate the expression levels of pro-inflammatory cytokines and is one of the key transcription factors regulating the transcription of pro-inflammatory cytokines (31). It is also a major substrate of various MAPK pathways (32). Under normal tissue conditions, basal FRA1 expression is low. However, FRA1 is overexpressed under inflammatory conditions (31). Low FOSL1 levels restrict the interaction of AP-1 with classical CRE and TRE elements (33).In chronic inflammatory diseases, NF-κB and FOSL1/AP-1 may be abnormally activated simultaneously, and their synergistic effect promotes the expression of some pro-inflammatory genes, leading to sustained pro-inflammatory gene expression, which may result in stronger inflammatory responses, thereby exacerbating disease progression (34). Furthermore, there is a complex interaction between NF-κB and FOSL1. NF-κB can directly regulate the expression of FOSL1 (35) or indirectly affect the activity of FOSL1 by regulating signaling pathways and the microenvironment (36). Moreover, FOSL1 is closely related to estrogen. Previous studies have shown that the expression of FOSL1 can be regulated by estrogen (37, 38). In estrogen receptor-positive (ER+) breast cancer cells, estrogen can directly or indirectly modulate FOSL1 expression through ER (39). High expression of FOSL1 is associated with poor prognosis in breast cancer, possibly by promoting epithelial-mesenchymal transition (EMT) and enhancing the migration and invasiveness of tumor cells (40). Additionally, FOSL1 can form heterodimers with other AP-1 family members (such as c-Jun, c-Fos, etc.) to regulate the expression of downstream genes, thereby participating in estrogen-mediated tumor growth and progression (41).

### 4.3 Upregulation of OSM damages the blood-brain barrier and exacerbates central inflammation

Oncostatin M (OSM) is a member of the IL-6 cytokine family and is involved in cell growth, differentiation, and inflammatory responses (42). OSM may either promote or suppress inflammation depending on the target cells and other cytokines present in the microenvironment. Its signaling can trigger the Ras-MAPK pathway, PI3K-Akt pathway, p38 and JNK MAPK pathways, and PKCδ activation. Moreover, OSM can cross the blood-brain barrier, where it contributes to the development of central nervous system diseases. In this process, OSM significantly downregulates the expression of adhesion molecules ICAM-1 and VCAM in vascular endothelial cells, reduces the function of tight junction proteins Claudin5 and E-cadherin, causing leakage of the blood-brain barrier and compromising its integrity. Subsequently, OSM acts on the OSMR of blood-brain barrier endothelial cells, promoting the secretion of the chemokine CCL20 by endothelial cells. CCL20 then activates Th17 cells through integrins α and promotes central immune infiltration of Th17 cells, exacerbating central inflammation (43). Simultaneously, OSM inhibits the uptake of glutamate by astrocytes through the JAK/STAT3 signaling pathway, leading to neuronal excitotoxicity (44). At the same time, OSM is closely related to estrogen. Studies have shown that in breast cancer, OSM interacts with estrogen signaling pathways, indirectly regulating the expression of estrogen receptors and promoting invasion and metastasis (45). OSM may also affect estrogen-related tumors by participating in the regulation of immune cells and inflammatory responses in the tumor microenvironment, and OSM is considered a potential therapeutic target (46). We found that among the various signaling pathways involved in the action of BSSGHY decoction, Cytokine-cytokine receptor interaction, PI3K-Akt signaling pathway, and JAK-STAT signaling pathway are closely related to OSM signaling expression. Furthermore, after treatment with BSSGHY decoction, the expression of FOS, NFKBIA, JUN, IL1B, RARA, IFNGR2, MAPK3, and IL4R genes was downregulated. These genes are closely related to Th17 cell differentiation, which to some extent indicates that BSSGHY decoction may achieve therapeutic effects by inhibiting Th17 cell differentiation, suppressing the expression of DEGs, thereby reducing central immune infiltration and alleviating central inflammation.

The results of this study, showing downregulation of FOSL1 and OSM after treatment with BSSGHY decoction, strongly suggest that during the perimenopausal period in women, alterations in estrogen-related pathways lead to upregulation of FOSL1 expression, mediating an increase in pro-inflammatory factors and dysregulation of immune cell function within the body (47), maintaining a state of chronic inflammation. Consequently, OSM is upregulated accordingly and crosses the blood-brain barrier, exacerbating central inflammation and leading to the occurrence of perimenopausal depression.

### 4.4 Upregulation of LINC01311 increases brain-derived estrogen levels, alleviates neuroinflammation, and consequently reduces impairment of neuroplasticity function

In our study, we found that there are many long non-coding RNAs (lncRNAs) among the DEGs. Over the past few decades, emerging evidence has suggested that lncRNAs may be abnormally expressed in the human brain and functionally participate in the development of neurodegenerative diseases, potentially serving as important functional regulators in the development and progression of neurodegenerative diseases (48). In this study, treatment with BSSGHY decoction resulted in significant upregulation of LINC01311, a novel lncRNA. It has been reported that the LINC01311/hsa-miR-146a-5p epigenetic axis is involved in the functional regulation of human lineage neurons in an Alzheimer’s disease cell model, and upregulation of LINC01311 has a protective effect against Aβ1-42-induced Alzheimer’s disease-like damage in SH-SY5Y cells (49). Furthermore, depression represents the most prominent psychiatric symptom associated with AD, and recent evidence suggests it may serve as a precursor or prodromal feature (50). Although the pathogenic mechanisms of depression and Alzheimer’s disease are not entirely consistent, depression is the most prominent psychiatric symptom associated with Alzheimer’s disease, and recent evidence suggests that depression may be a precursor or prodrome of Alzheimer’s disease (51). Therefore, we can speculate that brain-derived estrogen levels in perimenopausal depressed women may also be reduced to some extent, leading to impaired neuroplasticity function and neuroinflammation, thereby inducing depression. Following BSSGHY decoction intervention, LINC01311 upregulation further supports the involvement of this treatment in neuroplasticity modulation in perimenopausal depression. The mechanism may involve upregulating LINC01311 to increase brain-derived estrogen levels, alleviate neuroinflammation, and consequently reduce impairment of neuroplasticity function.

Undeniably, our study has some limitations. First, no healthy control group was included as a blank control, which may have limited the interpretation of the observed changes. Second, the results of the bioinformatics analysis were not further validated through *in vitro* or *in vivo* experiments, and thus, the underlying mechanisms remain to be confirmed. Third, the sample size was relatively small (n = 5), and in some cases, the differences between pre- and post-treatment samples were minimal. Future studies with larger cohorts and experimental validation are needed to strengthen the reliability and generalizability of these findings.

## 5 Conclusion

Our current study, through transcriptomic analysis, has revealed the crucial role of estrogen in perimenopausal depression through inflammatory and immune factor signaling, and elucidated the potential pathways, which may be the key pathways underlying the therapeutic effects of BSSGHY decoction. Additionally, it provides a hypothesis for the pathogenic mechanism of perimenopausal depression. This insight not only provides an important avenue for further exploring the mechanisms of BSSGHY decoction but also offers a valuable perspective and hypothesis for enhancing the prevention and treatment strategies for perimenopausal depression.

## Data Availability

The original contributions presented in the study are publicly available. This data can be found here: NCBI Gene Expression Omnibus, accession GSE308058.
