# Supplementary material for: Transcriptomic analysis reveals the key role of inflammatory and immune signaling in the anti-perimenopausal depression effects of Bushen Shugan Huayu decoction
Source: Front Psychiatry. 2025 Sep 26;16:1629900. doi: 10.3389/fpsyt.2025.1629900 (PMC12512047; doi:10.3389/fpsyt.2025.1629900)
Supplement: Supplementary file 1 [file Table1.docx]

**Table S1 Composition information of BSSGHY decoction**

| Botanical information | Pharmaceutical name | Chinese name | Dosage |
| --- | --- | --- | --- |
| The dried rhizome of Curculigo orchioides Gaertn. (Family: Amaryllidaceae) | Curculiginis Rhizoma | Xianmao  (仙茅) | 10g |
| The dried leaf of Epimedium brevicornu Maxim. (Family: Berberidaceae) | Epimedii Folium | Yinyanghuo  (淫羊藿) | 10g |
| The dried ripe fruit of Ligustrum lucidum Ait. (Family: Oleaceae) | Ligustri Lucidi Fructus | Nvzhenzi  (女贞子) | 20g |
| The dried aerial part of Eclipta prostrata L. (Family: Asteraceae) | Ecliptae Herba | Mohanlian  (墨旱莲) | 20g |
| The dried root of Bupleurum chinense DC. (Family: Apiaceae) | Bupleuri Radix | Chaihu  (柴胡) | 12g |
| The dried immature fruit of Citrus aurantium L. and its cultivars. (Family: Rutaceae) | Aurantii Fructus | Zhiqiao  (枳壳) | 6g |
| The dried rhizome of Ligusticum chuanxiong Hort. (Family: Apiaceae) | Chuanxiong Rhizoma | Chuanxiong  (川芎) | 12g |
| The dried body of Pheretima aspergillum(E. Perrier). (Family: Megascolecidae) | Pheretima | Dilong  (地龙) | 10g |

Administration: The above medicines were provided in granule form and taken twice daily after meals.
