# Supplementary material for: Transcriptomic analysis reveals the key role of inflammatory and immune signaling in the anti-perimenopausal depression effects of Bushen Shugan Huayu decoction
Source: Front Psychiatry. 2025 Sep 26;16:1629900. doi: 10.3389/fpsyt.2025.1629900 (PMC12512047; doi:10.3389/fpsyt.2025.1629900)
Supplement: Supplementary file 2 [file Table2.pdf]

Table S2 Summary of Differentially Expressed Genes (DEGs). The gene names (gene), the fold change (FoldChange), the significance of FC (sig_FC), the direction (Regulation), *p* value(*p*) , and the significance of *p* value (sig_*p*) of omics information are shown.

Table S2 Summary of Differentially Expressed Genes (DEGs)

Gene	FoldChange	sig_FC	*p*-value	sig_*p*	Regulation
LOC105374591	10.51	yes	5.81E-03	yes	Up
SAG	9.20	yes	3.80E-02	yes	Up
LOC105377134	9.14	yes	2.96E-02	yes	Up
PTGES2-AS1	8.37	yes	1.87E-02	yes	Up
FAM184B	8.07	yes	3.61E-02	yes	Up
LOC107986019	7.75	yes	4.59E-02	yes	Up
EFCAB6-DT	7.54	yes	4.70E-02	yes	Up
LOC105371953	7.48	yes	1.84E-02	yes	Up
LOC105372592	7.31	yes	2.62E-02	yes	Up
IL3RA	7.20	yes	1.42E-02	yes	Up
PBK	6.10	yes	1.75E-02	yes	Up
LOC101060400	5.58	yes	5.42E-03	yes	Up
ACTN1-DT	5.38	yes	2.05E-02	yes	Up
LOC105378326	5.08	yes	3.53E-02	yes	Up
CXCL3	5.01	yes	1.05E-02	yes	Up
CD99	4.97	yes	1.15E-02	yes	Up
PCDHGB3	4.95	yes	5.00E-03	yes	Up
DKK2	4.89	yes	2.78E-02	yes	Up
SLC26A7	4.86	yes	3.14E-02	yes	Up
CRYBB3	4.81	yes	1.70E-02	yes	Up
LOC105370518	4.80	yes	1.21E-02	yes	Up
LOC105372275	4.77	yes	2.03E-02	yes	Up
LOC105376675	4.74	yes	1.45E-02	yes	Up
CXCL11	4.31	yes	3.69E-02	yes	Up
CAVIN4	4.30	yes	2.93E-02	yes	Up
FGF2	3.90	yes	7.32E-03	yes	Up
FSIP2	3.38	yes	2.23E-02	yes	Up
LOC105378378	3.35	yes	1.79E-02	yes	Up
LOC105372026	3.33	yes	1.80E-02	yes	Up
LOC107985910	3.18	yes	4.79E-02	yes	Up
DNAI3	3.04	yes	3.72E-02	yes	Up
LOC105372952	3.04	yes	3.82E-03	yes	Up
LOC105373171	2.98	yes	2.69E-02	yes	Up
LOC107985306	2.98	yes	3.67E-02	yes	Up
HMMR	2.94	yes	1.15E-02	yes	Up
DPY19L2	2.84	yes	1.76E-02	yes	Up
ITFG2-AS1	2.70	yes	1.07E-02	yes	Up

L1TD1	2.66	yes	3.45E-03	yes	Up
GS1-124K5.4	2.65	yes	3.96E-02	yes	Up
LOC105376506	2.52	yes	4.27E-02	yes	Up
PDE3A	2.50	yes	5.25E-04	yes	Up
C3orf52	2.49	yes	4.20E-02	yes	Up
STK33	2.47	yes	9.34E-03	yes	Up
TBC1D8B	2.45	yes	2.92E-03	yes	Up
DISC1	2.44	yes	2.33E-02	yes	Up
XIRP2	2.37	yes	3.56E-02	yes	Up
NUDT6	2.36	yes	4.38E-03	yes	Up
FAM167B	2.28	yes	4.53E-02	yes	Up
LINC01311	2.28	yes	3.03E-02	yes	Up
LOC105373279	2.26	yes	1.40E-02	yes	Up
ADNP-AS1	2.20	yes	1.52E-02	yes	Up
FIRRE	2.20	yes	9.85E-03	yes	Up
PLOD2	2.18	yes	1.44E-02	yes	Up
SUCNR1	2.16	yes	1.01E-02	yes	Up
ALDH8A1	2.16	yes	1.47E-03	yes	Up
LOC105369901	2.13	yes	3.16E-02	yes	Up
MORF4L2-AS1	2.11	yes	2.65E-03	yes	Up
RALY-AS1	2.10	yes	3.86E-02	yes	Up
GTPBP6	2.10	yes	9.64E-03	yes	Up
POLQ	2.08	yes	9.38E-03	yes	Up
LINC01819	2.06	yes	1.66E-02	yes	Up
LOC105378248	2.05	yes	3.69E-03	yes	Up
LOC107986055	2.05	yes	1.99E-02	yes	Up
CNIH3	2.04	yes	2.51E-02	yes	Up
LOC102723446	2.04	yes	2.19E-04	yes	Up
RAD51AP1	2.01	yes	4.81E-03	yes	Up
LOC112268035	1.94	yes	1.32E-02	yes	Up
LOC105373730	1.92	yes	1.04E-03	yes	Up
LOC105374412	1.91	yes	8.92E-03	yes	Up
OXTR	1.90	yes	2.22E-03	yes	Up
OTUD7A	1.88	yes	5.76E-03	yes	Up
BFSP2-AS1	1.86	yes	8.34E-03	yes	Up
LINC00482	1.84	yes	1.20E-02	yes	Up
STXBP4	1.83	yes	9.52E-04	yes	Up
LOC105371773	1.83	yes	4.48E-02	yes	Up
LINC00944	1.83	yes	7.07E-03	yes	Up
CENPE	1.83	yes	1.19E-02	yes	Up
LOC105377548	1.81	yes	4.39E-02	yes	Up
WNT1	1.81	yes	4.60E-02	yes	Up
ZNF804A	1.80	yes	5.00E-02	yes	Up
XKRX	1.80	yes	6.19E-03	yes	Up

TSC22D1-AS1	1.80	yes	2.97E-02	yes	Up
TAS2R20	1.79	yes	2.37E-02	yes	Up
H2AC13	1.78	yes	3.34E-02	yes	Up
C3orf14	1.77	yes	4.11E-02	yes	Up
LOC107984140	1.77	yes	5.60E-03	yes	Up
PRTFDC1	1.77	yes	1.25E-02	yes	Up
LACC1	1.76	yes	8.23E-04	yes	Up
TMEM255A	1.76	yes	3.63E-02	yes	Up
LOC105375541	1.76	yes	3.76E-02	yes	Up
ATXN1-AS1	1.76	yes	3.32E-03	yes	Up
LOC105378948	1.76	yes	1.63E-02	yes	Up
P2RY1	1.75	yes	1.06E-05	yes	Up
KDM2B-DT	1.75	yes	4.65E-02	yes	Up
CCDC152	1.75	yes	1.19E-02	yes	Up
AKAP9	1.74	yes	1.01E-04	yes	Up
AKAP5	1.73	yes	9.47E-04	yes	Up
ATL1	1.72	yes	8.66E-04	yes	Up
MMRN1	1.72	yes	5.29E-03	yes	Up
TET1	1.72	yes	8.92E-03	yes	Up
ZNF502	1.71	yes	9.94E-03	yes	Up
NEB	1.70	yes	1.05E-02	yes	Up
KANSL1L-AS1	1.70	yes	7.30E-03	yes	Up
ZNF709	1.69	yes	2.44E-03	yes	Up
AGBL3	1.69	yes	8.80E-03	yes	Up
PLEKHH2	1.68	yes	4.61E-02	yes	Up
KIF20B	1.68	yes	9.08E-03	yes	Up
LOC101929174	1.66	yes	2.81E-02	yes	Up
RAP2C-AS1	1.66	yes	4.46E-02	yes	Up
DUS4L	1.65	yes	4.34E-02	yes	Up
LOC107986997	1.64	yes	2.57E-03	yes	Up
VEPH1	1.64	yes	1.23E-02	yes	Up
LINC01355	1.64	yes	3.74E-03	yes	Up
STON1	1.64	yes	9.24E-03	yes	Up
MAGI2-AS3	1.64	yes	7.09E-03	yes	Up
TENM1	1.64	yes	3.01E-03	yes	Up
CEP162	1.63	yes	7.70E-04	yes	Up
KLKB1	1.63	yes	2.14E-02	yes	Up
MRPL20-DT	1.63	yes	4.58E-02	yes	Up
PARD3	1.63	yes	4.66E-04	yes	Up
CEP128	1.63	yes	8.32E-03	yes	Up
RPGRIP1L	1.62	yes	4.20E-02	yes	Up
TRAPPC3L	1.62	yes	6.76E-03	yes	Up
BATF2	1.62	yes	2.56E-03	yes	Up
CEP290	1.62	yes	1.42E-03	yes	Up

ST8SIA6	1.62	yes	5.18E-03	yes	Up
SSPN	1.62	yes	1.71E-02	yes	Up
ARL17B	1.62	yes	3.88E-03	yes	Up
RAB27B	1.61	yes	1.22E-04	yes	Up
ETV7	1.61	yes	8.32E-03	yes	Up
CCNA2	1.61	yes	8.57E-03	yes	Up
LANCL3	1.61	yes	1.95E-02	yes	Up
LINC01259	1.60	yes	1.40E-02	yes	Up
JAKMIP2	1.60	yes	6.95E-04	yes	Up
BRCA2	1.60	yes	2.34E-02	yes	Up
LOC101928705	1.60	yes	1.30E-02	yes	Up
IRAK1BP1	1.60	yes	2.49E-02	yes	Up
DTHD1	1.59	yes	4.54E-04	yes	Up
ZNNT1	1.59	yes	3.06E-04	yes	Up
TSHZ2	1.59	yes	1.32E-03	yes	Up
HEMGN	1.58	yes	1.14E-03	yes	Up
ARHGEF2-AS2	1.58	yes	1.71E-02	yes	Up
EXTL2	1.58	yes	6.21E-03	yes	Up
DENND1B	1.58	yes	4.48E-03	yes	Up
DNM1	1.58	yes	4.04E-02	yes	Up
DYNC2H1	1.58	yes	2.60E-02	yes	Up
RFX3-DT	1.57	yes	8.74E-03	yes	Up
LINC01963	1.57	yes	3.02E-03	yes	Up
TTN	1.56	yes	2.94E-04	yes	Up
LOC105371224	1.56	yes	2.06E-02	yes	Up
LINC02246	1.56	yes	2.69E-02	yes	Up
ELOVL6	1.56	yes	5.41E-04	yes	Up
DNM3	1.56	yes	1.88E-04	yes	Up
ANGPT1	1.56	yes	5.37E-03	yes	Up
PCGF5	1.56	yes	1.30E-03	yes	Up
SLC6A4	1.55	yes	4.90E-02	yes	Up
NEXN	1.55	yes	5.55E-04	yes	Up
CIP2A	1.55	yes	2.76E-02	yes	Up
CD2AP	1.55	yes	1.44E-03	yes	Up
GOLGA4	1.55	yes	1.35E-03	yes	Up
LRRC1	1.55	yes	3.63E-02	yes	Up
PKHD1L1	1.53	yes	2.65E-03	yes	Up
CPLANE1	1.53	yes	3.09E-03	yes	Up
DDX60	1.53	yes	3.40E-03	yes	Up
GEN1	1.53	yes	9.78E-03	yes	Up
ZNF644	1.53	yes	2.78E-03	yes	Up
XRN1	1.53	yes	4.56E-03	yes	Up
TMEM254-AS1	1.52	yes	3.79E-02	yes	Up
RIF1	1.52	yes	3.01E-03	yes	Up

RBM41	1.52	yes	6.87E-03	yes	Up
ZNF680	1.52	yes	1.16E-02	yes	Up
MED12L	1.52	yes	1.90E-02	yes	Up
MAP10	1.51	yes	3.37E-02	yes	Up
SEPTIN4	1.51	yes	2.19E-02	yes	Up
ZBTB41	1.51	yes	1.58E-02	yes	Up
FAAP24	1.51	yes	4.27E-02	yes	Up
ZNF850	1.51	yes	1.17E-02	yes	Up
LOC105372446	1.51	yes	3.53E-03	yes	Up
SGO2	1.51	yes	4.02E-02	yes	Up
RAD50	1.50	yes	2.58E-03	yes	Up
LOC102723694	1.50	yes	1.65E-02	yes	Up
SLFN14	1.50	yes	6.38E-03	yes	Up
GBP1	1.50	yes	6.84E-03	yes	Up
ZC3HAV1L	1.50	yes	6.23E-03	yes	Up
CFH	1.50	yes	6.30E-03	yes	Up
C18orf54	1.50	yes	4.09E-02	yes	Up
C4orf50	1.49	yes	3.78E-02	yes	Up
GBP3	1.49	yes	7.02E-04	yes	Up
IGF2BP3	1.49	yes	8.23E-03	yes	Up
CCDC14	1.49	yes	2.74E-03	yes	Up
DGKH	1.49	yes	7.21E-03	yes	Up
VPS13A	1.49	yes	3.64E-03	yes	Up
KLRC4	1.48	yes	1.57E-02	yes	Up
CEP126	1.48	yes	2.72E-02	yes	Up
P2RY12	1.48	yes	1.12E-03	yes	Up
TBC1D32	1.48	yes	1.29E-02	yes	Up
LOC107986714	1.48	yes	1.79E-02	yes	Up
ZGRF1	1.48	yes	1.82E-02	yes	Up
SSX2IP	1.47	yes	4.91E-03	yes	Up
MAP9	1.47	yes	3.24E-02	yes	Up
ARAP2	1.47	yes	3.38E-03	yes	Up
TAS2R4	1.47	yes	2.33E-02	yes	Up
AFF2	1.47	yes	3.87E-02	yes	Up
KBTBD6	1.46	yes	1.76E-03	yes	Up
ATRX	1.46	yes	1.03E-02	yes	Up
IGIP	1.46	yes	1.88E-02	yes	Up
GXYLT1	1.46	yes	6.37E-03	yes	Up
BAG2	1.46	yes	8.05E-03	yes	Up
ZNF260	1.46	yes	1.16E-02	yes	Up
ATAD5	1.46	yes	9.63E-03	yes	Up
PMS1	1.46	yes	4.32E-03	yes	Up
ZNF845	1.46	yes	1.07E-02	yes	Up
FANCL	1.46	yes	1.95E-02	yes	Up

LINC00630	1.46	yes	4.27E-02	yes	Up
KIAA1109	1.46	yes	5.26E-03	yes	Up
CSPP1	1.45	yes	3.13E-03	yes	Up
AFAP1L2	1.45	yes	3.41E-02	yes	Up
MIR3142HG	1.45	yes	9.68E-03	yes	Up
DRICH1	1.45	yes	3.14E-02	yes	Up
BDP1	1.45	yes	5.13E-03	yes	Up
LCORL	1.45	yes	1.97E-02	yes	Up
MOB1B	1.45	yes	3.08E-03	yes	Up
MYZAP	1.44	yes	3.35E-02	yes	Up
LINC02555	1.44	yes	3.75E-02	yes	Up
ZBTB20	1.44	yes	1.15E-02	yes	Up
EPHA1-AS1	1.44	yes	3.19E-02	yes	Up
BEND2	1.44	yes	7.88E-04	yes	Up
AVPR1A	1.44	yes	4.93E-02	yes	Up
GUCY1B1	1.44	yes	2.87E-03	yes	Up
ZNF525	1.44	yes	1.68E-02	yes	Up
SACS	1.44	yes	9.69E-03	yes	Up
ZDHHC21	1.44	yes	2.61E-02	yes	Up
ZNF33B	1.44	yes	6.87E-03	yes	Up
ZNF470	1.44	yes	1.33E-02	yes	Up
MBNL3	1.44	yes	5.52E-03	yes	Up
SLK	1.44	yes	7.67E-03	yes	Up
DAAM1	1.44	yes	4.34E-03	yes	Up
UTRN	1.44	yes	3.80E-03	yes	Up
MSC-AS1	1.44	yes	4.29E-02	yes	Up
CNTRL	1.44	yes	2.41E-03	yes	Up
EXOC5	1.44	yes	6.40E-03	yes	Up
YES1	1.44	yes	2.06E-02	yes	Up
FKBP14	1.44	yes	4.79E-02	yes	Up
SYNE2	1.44	yes	3.45E-03	yes	Up
ZNF782	1.44	yes	1.79E-02	yes	Up
TRPC1	1.43	yes	3.46E-02	yes	Up
LOC105371461	1.43	yes	4.83E-02	yes	Up
SLC14A1	1.43	yes	1.41E-03	yes	Up
ESCO1	1.43	yes	2.49E-02	yes	Up
CEP350	1.43	yes	8.37E-03	yes	Up
FER	1.43	yes	3.88E-02	yes	Up
ENPP4	1.43	yes	1.35E-02	yes	Up
SPDL1	1.43	yes	8.57E-03	yes	Up
GAPT	1.43	yes	1.64E-02	yes	Up
TRIP11	1.43	yes	1.17E-02	yes	Up
MDS2	1.43	yes	2.05E-02	yes	Up
MANEA	1.43	yes	3.39E-02	yes	Up

CHM	1.43	yes	1.03E-02	yes	Up
LOC112267872	1.43	yes	4.25E-02	yes	Up
KATNAL1	1.42	yes	4.45E-03	yes	Up
CCP110	1.42	yes	1.49E-02	yes	Up
ARHGAP5	1.42	yes	8.69E-03	yes	Up
NRIP1	1.42	yes	8.93E-03	yes	Up
KBTBD8	1.42	yes	6.52E-03	yes	Up
VPS13C	1.42	yes	3.95E-03	yes	Up
SNAPC3	1.42	yes	2.04E-03	yes	Up
LMBRD2	1.42	yes	1.29E-02	yes	Up
RORA	1.42	yes	1.44E-02	yes	Up
NCKAP1	1.42	yes	3.56E-03	yes	Up
NEK1	1.42	yes	1.60E-02	yes	Up
SLFN5	1.42	yes	9.93E-03	yes	Up
SLC16A7	1.42	yes	2.98E-02	yes	Up
ZNF354A	1.42	yes	2.50E-03	yes	Up
TASOR2	1.42	yes	8.51E-04	yes	Up
TMEM81	1.42	yes	2.73E-02	yes	Up
NAA15	1.41	yes	9.04E-03	yes	Up
CCDC154	1.41	yes	3.65E-02	yes	Up
XAF1	1.41	yes	1.04E-02	yes	Up
ZNF546	1.41	yes	2.87E-02	yes	Up
TNNT3	1.41	yes	4.77E-02	yes	Up
TFEC	1.41	yes	2.29E-03	yes	Up
ATP11C	1.41	yes	8.02E-03	yes	Up
UFL1	1.41	yes	1.24E-02	yes	Up
NPAT	1.41	yes	1.24E-02	yes	Up
ZNF718	1.41	yes	2.81E-03	yes	Up
SHPRH	1.41	yes	4.72E-03	yes	Up
MYO6	1.41	yes	4.83E-02	yes	Up
APOL6	1.41	yes	7.25E-03	yes	Up
SMARCAD1	1.41	yes	1.48E-02	yes	Up
ZMAT1	1.41	yes	1.17E-02	yes	Up
RANBP2	1.41	yes	9.00E-03	yes	Up
NCOA7	1.41	yes	3.45E-03	yes	Up
ZNF100	1.41	yes	1.64E-02	yes	Up
UPF3B	1.40	yes	5.22E-03	yes	Up
ZNF235	1.40	yes	8.20E-03	yes	Up
ZNF302	1.40	yes	3.87E-03	yes	Up
PUS7L	1.40	yes	2.06E-02	yes	Up
IKZF2	1.40	yes	1.54E-02	yes	Up
USPL1	1.40	yes	2.90E-03	yes	Up
PWAR6	1.40	yes	4.64E-02	yes	Up
MKI67	1.40	yes	2.73E-02	yes	Up

ZNF528	1.40	yes	6.55E-03	yes	Up
MBNL2	1.40	yes	2.28E-02	yes	Up
CHD9	1.40	yes	1.33E-02	yes	Up
USP47	1.40	yes	7.16E-03	yes	Up
CALD1	1.40	yes	3.46E-02	yes	Up
CASP8AP2	1.40	yes	1.42E-02	yes	Up
TRIM23	1.40	yes	6.02E-03	yes	Up
CLDND2	1.40	yes	7.47E-04	yes	Up
LIG4	1.40	yes	1.72E-02	yes	Up
EEA1	1.40	yes	2.81E-02	yes	Up
ZNF615	1.40	yes	1.41E-02	yes	Up
ALG10B	1.40	yes	3.58E-02	yes	Up
VPS50	1.39	yes	1.53E-02	yes	Up
ZNF594	1.39	yes	1.44E-02	yes	Up
CLOCK	1.39	yes	1.19E-02	yes	Up
GPRIN3	1.39	yes	1.40E-02	yes	Up
KIF21A	1.39	yes	5.81E-03	yes	Up
NHLRC2	1.39	yes	1.63E-02	yes	Up
ZNF28	1.39	yes	1.48E-02	yes	Up
KLRF1	1.39	yes	1.15E-02	yes	Up
SASS6	1.39	yes	4.13E-02	yes	Up
MIS18BP1	1.39	yes	1.48E-02	yes	Up
TTC14	1.39	yes	9.83E-03	yes	Up
PHLDB2	1.39	yes	7.70E-03	yes	Up
ESF1	1.39	yes	3.45E-02	yes	Up
PTPN4	1.39	yes	1.36E-02	yes	Up
ZNF780B	1.39	yes	9.70E-03	yes	Up
ENKUR	1.39	yes	1.94E-03	yes	Up
SMC4	1.39	yes	2.89E-02	yes	Up
PSTPIP2	1.39	yes	2.53E-02	yes	Up
UEVLD	1.38	yes	1.15E-02	yes	Up
KLF12	1.38	yes	1.70E-02	yes	Up
ZNF92	1.38	yes	3.43E-02	yes	Up
IL6ST	1.38	yes	1.92E-02	yes	Up
IFIH1	1.38	yes	1.50E-02	yes	Up
THOC2	1.38	yes	7.07E-03	yes	Up
CEP78	1.38	yes	1.48E-02	yes	Up
LRRC40	1.38	yes	1.72E-02	yes	Up
PTPN13	1.38	yes	1.37E-02	yes	Up
FMO4	1.38	yes	3.33E-02	yes	Up
KTN1	1.38	yes	1.48E-02	yes	Up
BIRC3	1.38	yes	2.59E-02	yes	Up
BRCA1	1.38	yes	2.71E-02	yes	Up
ATR	1.38	yes	9.13E-03	yes	Up

RSF1	1.38	yes	1.66E-02	yes	Up
LINC00987	1.38	yes	3.94E-02	yes	Up
SCML1	1.38	yes	3.34E-02	yes	Up
SMC3	1.38	yes	1.10E-02	yes	Up
TMEM161B	1.38	yes	8.74E-03	yes	Up
LRP12	1.38	yes	3.81E-02	yes	Up
ERAP2	1.38	yes	2.55E-03	yes	Up
EIF5B	1.38	yes	3.15E-02	yes	Up
CD226	1.38	yes	6.65E-03	yes	Up
DNAJB14	1.38	yes	3.22E-02	yes	Up
CUL5	1.38	yes	2.00E-02	yes	Up
KPNA5	1.38	yes	4.62E-02	yes	Up
UTP23	1.38	yes	2.49E-02	yes	Up
GCC2	1.38	yes	3.43E-02	yes	Up
ZNF605	1.38	yes	2.55E-02	yes	Up
STARD4	1.38	yes	7.50E-03	yes	Up
CDK6	1.38	yes	1.24E-02	yes	Up
CWF19L2	1.38	yes	9.61E-03	yes	Up
FKTN	1.37	yes	2.64E-02	yes	Up
MEST	1.37	yes	2.80E-02	yes	Up
NKTR	1.37	yes	7.88E-03	yes	Up
GASK1B-AS1	1.37	yes	1.37E-02	yes	Up
BRWD1	1.37	yes	2.39E-02	yes	Up
ROCK2	1.37	yes	2.27E-02	yes	Up
ANKEF1	1.37	yes	1.74E-02	yes	Up
FPGT	1.37	yes	1.87E-02	yes	Up
GOLGB1	1.37	yes	1.74E-02	yes	Up
SRFBP1	1.37	yes	4.73E-02	yes	Up
STON2	1.37	yes	1.58E-02	yes	Up
LOC105369477	1.37	yes	4.83E-02	yes	Up
CHD1	1.37	yes	2.26E-02	yes	Up
SSB	1.37	yes	1.80E-02	yes	Up
VRK2	1.37	yes	2.05E-02	yes	Up
IBTK	1.37	yes	2.58E-02	yes	Up
SPEF2	1.37	yes	4.38E-02	yes	Up
PEX3	1.37	yes	3.21E-02	yes	Up
PDE5A	1.36	yes	4.80E-03	yes	Up
MPHOSPH10	1.36	yes	1.37E-02	yes	Up
ZNF273	1.36	yes	3.45E-02	yes	Up
KLRC3	1.36	yes	8.32E-03	yes	Up
LOC105371870	1.36	yes	1.30E-02	yes	Up
RECQL	1.36	yes	8.87E-03	yes	Up
TMF1	1.36	yes	2.24E-02	yes	Up
SOCS4	1.36	yes	2.93E-02	yes	Up

LUC7L3	1.36	yes	5.01E-03	yes	Up
ZNF227	1.36	yes	7.08E-03	yes	Up
CTNNAL1	1.36	yes	4.35E-02	yes	Up
ASAP2	1.36	yes	2.23E-02	yes	Up
ZNF600	1.36	yes	4.02E-03	yes	Up
CCDC186	1.36	yes	3.78E-02	yes	Up
PIBF1	1.36	yes	2.73E-02	yes	Up
GUCY1A1	1.36	yes	1.31E-02	yes	Up
CAMK4	1.36	yes	2.97E-02	yes	Up
THUMPD3-AS1	1.36	yes	1.26E-02	yes	Up
NUP43	1.36	yes	1.46E-03	yes	Up
CCDC66	1.36	yes	2.76E-02	yes	Up
PURA	1.36	yes	8.92E-03	yes	Up
TRMT13	1.35	yes	3.62E-02	yes	Up
FAM126A	1.35	yes	1.30E-02	yes	Up
LRRC58	1.35	yes	4.88E-02	yes	Up
MYSM1	1.35	yes	2.92E-02	yes	Up
AGL	1.35	yes	2.29E-02	yes	Up
LOC107985876	1.35	yes	3.11E-03	yes	Up
ITGAV	1.35	yes	1.27E-02	yes	Up
DNM1L	1.35	yes	6.86E-03	yes	Up
CEP85L	1.35	yes	2.95E-02	yes	Up
LOC105371661	1.35	yes	1.18E-02	yes	Up
CD274	1.35	yes	4.35E-02	yes	Up
ZNF654	1.35	yes	3.30E-02	yes	Up
SESTD1	1.35	yes	1.16E-02	yes	Up
OTUD6B-AS1	1.35	yes	4.60E-02	yes	Up
PRKACB	1.35	yes	3.06E-02	yes	Up
KRIT1	1.35	yes	1.71E-02	yes	Up
PCM1	1.35	yes	1.66E-02	yes	Up
FAM135A	1.35	yes	1.72E-02	yes	Up
RGS1	1.35	yes	3.97E-02	yes	Up
PIK3C2A	1.35	yes	3.32E-02	yes	Up
MTREX	1.35	yes	1.23E-02	yes	Up
PANK3	1.35	yes	1.71E-02	yes	Up
MACF1	1.35	yes	2.61E-03	yes	Up
LSM8	1.35	yes	3.37E-02	yes	Up
GPAM	1.35	yes	2.11E-02	yes	Up
TMX3	1.35	yes	1.61E-02	yes	Up
REL	1.35	yes	2.51E-02	yes	Up
POLK	1.35	yes	1.72E-02	yes	Up
ZNF354C	1.35	yes	2.77E-02	yes	Up
DAB2	1.35	yes	1.73E-03	yes	Up
USP34	1.35	yes	9.84E-03	yes	Up

INPP4B	1.35	yes	2.87E-02	yes	Up
FCRL5	1.34	yes	2.02E-02	yes	Up
CAVIN2	1.34	yes	1.53E-03	yes	Up
MYEF2	1.34	yes	3.08E-02	yes	Up
ENPP5	1.34	yes	4.78E-02	yes	Up
KIF2A	1.34	yes	1.02E-02	yes	Up
ERCC6L2	1.34	yes	2.73E-02	yes	Up
GBP4	1.34	yes	3.15E-02	yes	Up
ZNF292	1.34	yes	3.95E-02	yes	Up
SCAF11	1.34	yes	3.01E-02	yes	Up
PDIK1L	1.34	yes	2.56E-02	yes	Up
HCFC2	1.34	yes	1.77E-02	yes	Up
ZC3H13	1.34	yes	1.69E-02	yes	Up
ANKRD26	1.34	yes	3.64E-02	yes	Up
FMN1	1.34	yes	3.17E-02	yes	Up
PLCB1	1.34	yes	4.03E-02	yes	Up
KLRC2	1.34	yes	2.28E-02	yes	Up
CEP295	1.34	yes	3.04E-02	yes	Up
BBX	1.34	yes	3.60E-02	yes	Up
SLC39A10	1.34	yes	1.80E-02	yes	Up
ANKRD36C	1.34	yes	3.38E-02	yes	Up
AMY2B	1.34	yes	1.90E-02	yes	Up
LINC00861	1.34	yes	1.70E-02	yes	Up
ATM	1.34	yes	2.20E-02	yes	Up
U2SURP	1.34	yes	3.65E-02	yes	Up
JRKL	1.34	yes	2.46E-02	yes	Up
DOP1A	1.34	yes	8.42E-03	yes	Up
TCAF1	1.33	yes	3.12E-02	yes	Up
TYW5	1.33	yes	3.33E-02	yes	Up
DNAJC13	1.33	yes	1.15E-02	yes	Up
ANK3	1.33	yes	2.26E-02	yes	Up
PARP14	1.33	yes	2.55E-02	yes	Up
ZFC3H1	1.33	yes	2.59E-02	yes	Up
RPAP2	1.33	yes	3.07E-02	yes	Up
ZFP14	1.33	yes	1.55E-02	yes	Up
LEPR	1.33	yes	4.17E-02	yes	Up
LINC02648	1.33	yes	2.18E-02	yes	Up
PNPT1	1.33	yes	2.61E-02	yes	Up
ZHX1	1.33	yes	2.57E-02	yes	Up
ZNF10	1.33	yes	4.95E-02	yes	Up
RO60	1.33	yes	3.54E-02	yes	Up
USP16	1.33	yes	2.83E-02	yes	Up
ZRANB2	1.33	yes	3.19E-02	yes	Up
C2orf88	1.33	yes	1.74E-03	yes	Up

RESF1	1.33	yes	4.06E-02	yes	Up
BICD1	1.33	yes	4.48E-02	yes	Up
RFC1	1.33	yes	2.21E-02	yes	Up
LARP4	1.33	yes	2.12E-02	yes	Up
TOP2B	1.33	yes	2.74E-02	yes	Up
CCDC82	1.33	yes	3.85E-02	yes	Up
LHFPL2	1.33	yes	4.27E-02	yes	Up
NIPBL	1.33	yes	3.85E-02	yes	Up
ARHGAP18	1.33	yes	1.40E-02	yes	Up
ARL13B	1.33	yes	3.10E-02	yes	Up
OPA1	1.33	yes	1.77E-02	yes	Up
CWC22	1.33	yes	4.46E-02	yes	Up
SMG1	1.32	yes	9.82E-03	yes	Up
FRYL	1.32	yes	1.71E-02	yes	Up
MAN1A2	1.32	yes	3.90E-02	yes	Up
GUF1	1.32	yes	2.92E-02	yes	Up
GIPR	1.32	yes	1.92E-02	yes	Up
PNMA3	1.32	yes	4.45E-02	yes	Up
RGS18	1.32	yes	3.06E-02	yes	Up
ARHGAP21	1.32	yes	5.67E-03	yes	Up
DMXL1	1.32	yes	3.14E-02	yes	Up
GOLIM4	1.32	yes	2.73E-02	yes	Up
HELB	1.32	yes	4.48E-03	yes	Up
GCLM	1.32	yes	1.07E-02	yes	Up
ZNF800	1.32	yes	3.04E-02	yes	Up
ZNF638	1.32	yes	3.95E-02	yes	Up
CCDC91	1.32	yes	4.95E-02	yes	Up
ZNF37A	1.32	yes	4.39E-02	yes	Up
ZNF518A	1.32	yes	4.47E-02	yes	Up
DENND4A	1.32	yes	1.88E-02	yes	Up
CDC14B	1.32	yes	8.58E-03	yes	Up
ETNK1	1.32	yes	3.47E-02	yes	Up
FAM111A	1.32	yes	9.01E-03	yes	Up
ZNF721	1.32	yes	4.49E-02	yes	Up
SLFN12L	1.32	yes	2.00E-02	yes	Up
LINC01138	1.32	yes	4.33E-03	yes	Up
MEIS1	1.32	yes	1.61E-02	yes	Up
NEMF	1.32	yes	3.65E-02	yes	Up
PGGT1B	1.32	yes	4.28E-02	yes	Up
SMC5	1.32	yes	3.22E-02	yes	Up
CMTM1	1.32	yes	4.40E-02	yes	Up
HSPH1	1.31	yes	1.46E-02	yes	Up
SMC6	1.31	yes	4.73E-02	yes	Up
ACADSB	1.31	yes	1.86E-02	yes	Up

CBWD5	1.31	yes	3.64E-02	yes	Up
DPH6	1.31	yes	4.62E-02	yes	Up
DHX36	1.31	yes	2.98E-02	yes	Up
SETX	1.31	yes	2.53E-02	yes	Up
ABCA5	1.31	yes	3.08E-02	yes	Up
C21orf91	1.31	yes	4.94E-02	yes	Up
PNISR	1.31	yes	2.62E-02	yes	Up
AIM2	1.31	yes	3.66E-02	yes	Up
SYNE1	1.31	yes	5.20E-03	yes	Up
RPAP3	1.31	yes	3.54E-02	yes	Up
MAP4K5	1.31	yes	3.04E-02	yes	Up
N4BP2	1.31	yes	4.72E-02	yes	Up
ZNF107	1.31	yes	4.68E-02	yes	Up
ZNF507	1.31	yes	3.33E-02	yes	Up
TPR	1.31	yes	3.46E-02	yes	Up
PYHIN1	1.31	yes	2.35E-02	yes	Up
ZFP1	1.31	yes	4.14E-02	yes	Up
RNPC3	1.31	yes	1.66E-02	yes	Up
LIMS1	1.31	yes	1.04E-02	yes	Up
CARF	1.31	yes	1.70E-02	yes	Up
UTP15	1.31	yes	2.66E-02	yes	Up
LOC105370792	1.31	yes	2.68E-02	yes	Up
TYW3	1.31	yes	4.67E-02	yes	Up
KNTC1	1.31	yes	2.07E-02	yes	Up
PHACTR2	1.31	yes	4.08E-02	yes	Up
RBM26	1.31	yes	3.13E-02	yes	Up
PIGF	1.31	yes	2.27E-02	yes	Up
NOL8	1.31	yes	3.08E-02	yes	Up
ANKRD36	1.31	yes	3.55E-02	yes	Up
UBA6	1.31	yes	4.50E-02	yes	Up
EHBP1	1.31	yes	2.95E-02	yes	Up
ZBTB6	1.31	yes	3.31E-02	yes	Up
RTP4	1.31	yes	3.53E-02	yes	Up
SCAPER	1.31	yes	4.19E-02	yes	Up
PHC3	1.31	yes	3.35E-02	yes	Up
RNF6	1.31	yes	3.42E-02	yes	Up
USO1	1.30	yes	2.84E-02	yes	Up
LOC105375754	1.30	yes	2.71E-02	yes	Up
PRPF40A	1.30	yes	4.69E-02	yes	Up
ATP8A1	1.30	yes	4.18E-02	yes	Up
NBEAL1	1.30	yes	1.50E-02	yes	Up
ATAD2B	1.30	yes	1.84E-02	yes	Up
ZNF181	1.30	yes	4.52E-02	yes	Up
ZBTB38	1.30	yes	2.55E-02	yes	Up

TTC3	1.30	yes	3.00E-02	yes	Up
AP4E1	1.30	yes	4.17E-02	yes	Up
TRNT1	1.30	yes	2.79E-02	yes	Up
GPR171	1.30	yes	4.39E-02	yes	Up
ITPR2	1.30	yes	2.18E-02	yes	Up
DCLRE1C	1.30	yes	1.27E-02	yes	Up
RBAK	1.30	yes	4.09E-02	yes	Up
MYO9A	1.30	yes	3.37E-02	yes	Up
DEK	1.30	yes	4.80E-02	yes	Up
FRMD4B	1.30	yes	9.59E-03	yes	Up
PRPF4B	1.30	yes	1.59E-02	yes	Up
ZNF141	1.30	yes	3.32E-02	yes	Up
PIK3R1	1.30	yes	1.91E-02	yes	Up
SLC35A3	1.30	yes	3.09E-02	yes	Up
AKAP11	1.30	yes	3.68E-02	yes	Up
SOS1	1.30	yes	2.07E-02	yes	Up
ITGA4	1.30	yes	3.64E-02	yes	Up
PAXBP1	1.30	yes	3.31E-02	yes	Up
LRBA	1.30	yes	2.58E-03	yes	Up
UBA5	1.30	yes	4.12E-02	yes	Up
USP8	1.30	yes	4.32E-02	yes	Up
DIS3	1.30	yes	3.73E-02	yes	Up
KCNA3	1.29	yes	1.01E-02	yes	Up
LOC102724808	1.29	yes	2.04E-02	yes	Up
TTC37	1.29	yes	3.49E-02	yes	Up
TRIM59	1.29	yes	4.50E-02	yes	Up
DBT	1.29	yes	2.87E-02	yes	Up
TIA1	1.29	yes	2.40E-02	yes	Up
OFD1	1.29	yes	2.16E-02	yes	Up
RUFY2	1.29	yes	4.74E-02	yes	Up
ADAM28	1.29	yes	3.42E-02	yes	Up
OXR1	1.29	yes	4.42E-02	yes	Up
LEO1	1.29	yes	2.56E-02	yes	Up
MYBL1	1.29	yes	3.31E-02	yes	Up
AGAP9	1.29	yes	1.78E-02	yes	Up
FASLG	1.29	yes	2.10E-02	yes	Up
PTPN22	1.29	yes	2.12E-02	yes	Up
MITD1	1.29	yes	3.34E-02	yes	Up
MBNL1	1.29	yes	3.72E-02	yes	Up
NT5C3A	1.29	yes	2.62E-02	yes	Up
MARCHF1	1.28	yes	3.15E-02	yes	Up
LSM11	1.28	yes	2.67E-02	yes	Up
KLRG1	1.28	yes	5.78E-03	yes	Up
GPR174	1.28	yes	3.46E-02	yes	Up

MYCBP2	1.28	yes	1.21E-02	yes	Up
KIAA2026	1.28	yes	2.81E-02	yes	Up
IQCG	1.28	yes	4.98E-02	yes	Up
ZNF121	1.28	yes	4.91E-02	yes	Up
UBR1	1.28	yes	2.46E-02	yes	Up
CLEC2D	1.28	yes	4.05E-02	yes	Up
THEMIS	1.28	yes	4.35E-02	yes	Up
ZNF234	1.28	yes	2.75E-02	yes	Up
TC2N	1.28	yes	3.70E-02	yes	Up
LOC107987222	1.28	yes	1.99E-02	yes	Up
TASOR	1.28	yes	4.25E-02	yes	Up
ZNF83	1.28	yes	3.38E-02	yes	Up
COG6	1.28	yes	2.62E-02	yes	Up
SCRN3	1.28	yes	4.08E-02	yes	Up
ICE1	1.28	yes	2.82E-02	yes	Up
NR1D2	1.28	yes	4.37E-02	yes	Up
WRN	1.28	yes	3.98E-02	yes	Up
PPP2R2B	1.28	yes	4.28E-02	yes	Up
JAK2	1.28	yes	3.64E-02	yes	Up
TRPM7	1.28	yes	3.89E-02	yes	Up
UBN2	1.28	yes	3.48E-02	yes	Up
TARP	1.28	yes	3.89E-02	yes	Up
ZNF12	1.28	yes	4.41E-02	yes	Up
EPRS1	1.28	yes	2.25E-02	yes	Up
ELOVL7	1.28	yes	2.91E-02	yes	Up
PLA2G12A	1.28	yes	1.22E-02	yes	Up
LNPEP	1.27	yes	3.90E-02	yes	Up
CFAP97	1.27	yes	3.48E-02	yes	Up
TTC33	1.27	yes	3.99E-02	yes	Up
LPAR6	1.27	yes	1.98E-02	yes	Up
MIA3	1.27	yes	3.00E-02	yes	Up
RBM27	1.27	yes	4.92E-02	yes	Up
GTF3C3	1.27	yes	2.67E-02	yes	Up
DMTF1	1.27	yes	3.87E-02	yes	Up
RPE	1.27	yes	2.96E-02	yes	Up
ELK4	1.27	yes	2.47E-02	yes	Up
RANBP6	1.27	yes	4.58E-02	yes	Up
YTHDC2	1.27	yes	3.67E-02	yes	Up
DENND4C	1.27	yes	1.42E-02	yes	Up
CENPJ	1.27	yes	4.61E-02	yes	Up
SMIM13	1.27	yes	3.78E-02	yes	Up
KIFAP3	1.27	yes	3.91E-02	yes	Up
GIMAP7	1.27	yes	9.96E-03	yes	Up
DDHD1	1.27	yes	4.41E-02	yes	Up

ZMYM4	1.27	yes	3.36E-02	yes	Up
SP140	1.27	yes	2.58E-02	yes	Up
RBM25	1.27	yes	4.24E-02	yes	Up
CUL2	1.27	yes	3.91E-02	yes	Up
AZI2	1.27	yes	2.84E-02	yes	Up
CROT	1.27	yes	3.37E-02	yes	Up
DNAJC10	1.26	yes	3.30E-02	yes	Up
C2CD5	1.26	yes	4.61E-02	yes	Up
TRIM22	1.26	yes	4.15E-02	yes	Up
ARFGEF1	1.26	yes	3.74E-02	yes	Up
DCAF17	1.26	yes	3.68E-02	yes	Up
PIKFYVE	1.26	yes	4.16E-02	yes	Up
ZBTB26	1.26	yes	4.39E-02	yes	Up
TUT4	1.26	yes	2.87E-02	yes	Up
ASH1L	1.26	yes	2.53E-02	yes	Up
BPTF	1.26	yes	3.30E-02	yes	Up
GAN	1.26	yes	2.58E-02	yes	Up
PARP11	1.26	yes	3.83E-02	yes	Up
ARHGEF12	1.26	yes	3.94E-02	yes	Up
ANKIB1	1.26	yes	2.41E-02	yes	Up
CCAR1	1.26	yes	3.00E-02	yes	Up
TMOD2	1.26	yes	4.42E-02	yes	Up
MGA	1.25	yes	2.86E-02	yes	Up
AP5M1	1.25	yes	3.42E-02	yes	Up
RAB3GAP2	1.25	yes	4.98E-02	yes	Up
PDS5A	1.25	yes	4.97E-02	yes	Up
ZNF766	1.25	yes	4.35E-02	yes	Up
TTC21B	1.25	yes	2.94E-02	yes	Up
WDFY1	1.25	yes	4.89E-02	yes	Up
SECISBP2L	1.25	yes	4.23E-02	yes	Up
BIRC6	1.25	yes	2.74E-02	yes	Up
METAP2	1.25	yes	3.07E-02	yes	Up
MIAT	1.25	yes	1.27E-02	yes	Up
SYTL2	1.25	yes	3.08E-02	yes	Up
BLOC1S6	1.25	yes	4.04E-02	yes	Up
GNPTAB	1.25	yes	2.16E-02	yes	Up
DOCK10	1.25	yes	4.17E-02	yes	Up
NAA35	1.25	yes	3.88E-02	yes	Up
SRSF11	1.25	yes	4.61E-02	yes	Up
SBNO1	1.25	yes	2.89E-02	yes	Up
SLF2	1.25	yes	4.63E-02	yes	Up
SHTN1	1.25	yes	1.44E-02	yes	Up
ABCB1	1.25	yes	4.09E-02	yes	Up
LOC286437	1.25	yes	4.54E-02	yes	Up

POU2F1	1.25	yes	4.52E-02	yes	Up
RASA1	1.25	yes	3.93E-02	yes	Up
IPO7	1.25	yes	4.46E-02	yes	Up
PTPRM	1.25	yes	3.95E-02	yes	Up
EML4	1.24	yes	3.52E-02	yes	Up
TMEM184C	1.24	yes	2.85E-02	yes	Up
BTAF1	1.24	yes	1.79E-02	yes	Up
XIST	1.24	yes	4.05E-02	yes	Up
SKP2	1.24	yes	4.03E-02	yes	Up
ZFP62	1.24	yes	3.12E-02	yes	Up
RHOU	1.24	yes	2.81E-02	yes	Up
ADAT2	1.24	yes	2.50E-02	yes	Up
IREB2	1.24	yes	4.98E-02	yes	Up
CAND1	1.24	yes	2.82E-02	yes	Up
NIT2	1.24	yes	4.21E-02	yes	Up
ZC3H14	1.24	yes	3.62E-02	yes	Up
KLHL3	1.24	yes	4.09E-02	yes	Up
STX17	1.24	yes	4.70E-02	yes	Up
R3HCC1L	1.24	yes	3.94E-02	yes	Up
ZNF841	1.24	yes	4.95E-02	yes	Up
SLAMF7	1.24	yes	1.00E-02	yes	Up
CPSF6	1.23	yes	2.81E-02	yes	Up
GK5	1.23	yes	4.59E-02	yes	Up
HEATR1	1.23	yes	2.52E-02	yes	Up
CCDC18-AS1	1.23	yes	2.86E-02	yes	Up
SLC26A2	1.23	yes	4.19E-02	yes	Up
ALG13	1.23	yes	4.27E-02	yes	Up
MFAP3L	1.23	yes	2.59E-02	yes	Up
TGS1	1.23	yes	3.17E-02	yes	Up
EIF3A	1.23	yes	4.31E-02	yes	Up
PRR14L	1.23	yes	4.82E-02	yes	Up
DNAJC15	1.23	yes	4.62E-02	yes	Up
SEPTIN11	1.23	yes	3.92E-02	yes	Up
CD96	1.23	yes	2.55E-02	yes	Up
GSAP	1.23	yes	2.30E-02	yes	Up
BTN3A1	1.22	yes	4.74E-02	yes	Up
RABEP1	1.22	yes	4.96E-02	yes	Up
TTC13	1.22	yes	3.65E-02	yes	Up
PREPL	1.22	yes	4.14E-02	yes	Up
TM2D1	1.22	yes	4.73E-02	yes	Up
FUBP1	1.22	yes	2.23E-02	yes	Up
GARS1-DT	1.22	yes	4.45E-02	yes	Up
PPM1L	1.22	yes	4.18E-02	yes	Up
PARP15	1.22	yes	3.21E-02	yes	Up

LRRC8C	1.22	yes	4.25E-02	yes	Up
SP140L	1.22	yes	3.86E-02	yes	Up
TNRC6A	1.22	yes	3.21E-02	yes	Up
ZKSCAN8	1.21	yes	4.18E-02	yes	Up
GOLGA8A	1.21	yes	4.76E-02	yes	Up
NUP107	1.21	yes	4.64E-02	yes	Up
SENP1	1.21	yes	4.38E-02	yes	Up
F2R	1.21	yes	3.29E-02	yes	Up
PATL2	1.20	yes	3.70E-02	yes	Up
CCR1	0.83	yes	4.47E-02	yes	Down
SRXN1	0.83	yes	4.56E-02	yes	Down
PPP1R9B	0.83	yes	4.79E-02	yes	Down
COQ8A	0.83	yes	3.44E-02	yes	Down
CNPY3	0.83	yes	4.49E-02	yes	Down
SVIL	0.83	yes	2.86E-02	yes	Down
VAV1	0.83	yes	4.29E-02	yes	Down
POLR2C	0.83	yes	3.04E-02	yes	Down
C5AR2	0.83	yes	3.20E-02	yes	Down
MYO1F	0.83	yes	4.70E-02	yes	Down
TPRG1L	0.83	yes	3.74E-02	yes	Down
MXD3	0.83	yes	4.37E-02	yes	Down
DAD1	0.83	yes	4.28E-02	yes	Down
S100A8	0.83	yes	3.37E-02	yes	Down
CBFA2T3	0.83	yes	4.45E-02	yes	Down
FCGRT	0.83	yes	4.96E-02	yes	Down
FKBP8	0.83	yes	4.62E-02	yes	Down
CLIC1	0.83	yes	3.85E-02	yes	Down
SRA1	0.83	yes	4.72E-02	yes	Down
SELENOK	0.83	yes	3.24E-02	yes	Down
PBXIP1	0.83	yes	2.97E-02	yes	Down
NCF2	0.83	yes	2.69E-02	yes	Down
IL1RN	0.83	yes	4.24E-02	yes	Down
ANXA11	0.83	yes	3.40E-02	yes	Down
TRMT112	0.82	yes	4.65E-02	yes	Down
RNF167	0.82	yes	3.87E-02	yes	Down
LAMTOR5	0.82	yes	2.55E-02	yes	Down
F11R	0.82	yes	4.40E-02	yes	Down
RELT	0.82	yes	3.89E-02	yes	Down
RNF149	0.82	yes	3.79E-02	yes	Down
FRMD8	0.82	yes	4.19E-02	yes	Down
MRPL49	0.82	yes	4.76E-02	yes	Down
SRGN	0.82	yes	4.41E-02	yes	Down
MID1IP1	0.82	yes	1.74E-02	yes	Down
PPP1R10	0.82	yes	2.10E-02	yes	Down

C3orf62	0.82	yes	3.97E-02	yes	Down
PNRC1	0.82	yes	1.85E-02	yes	Down
TYROBP	0.82	yes	4.50E-02	yes	Down
APMAP	0.82	yes	3.16E-02	yes	Down
H3-3B	0.82	yes	3.03E-02	yes	Down
ZNF746	0.82	yes	3.09E-02	yes	Down
NOP10	0.82	yes	1.58E-02	yes	Down
RNF19B	0.82	yes	3.23E-02	yes	Down
GAB2	0.82	yes	3.04E-02	yes	Down
ARRB2	0.82	yes	4.65E-02	yes	Down
UBC	0.82	yes	3.08E-02	yes	Down
STK16	0.82	yes	4.00E-02	yes	Down
RAB5C	0.82	yes	3.96E-02	yes	Down
TNIP1	0.82	yes	2.18E-02	yes	Down
BID	0.82	yes	3.41E-02	yes	Down
WDFY4	0.82	yes	4.39E-02	yes	Down
RCN3	0.82	yes	3.43E-02	yes	Down
STAT5B	0.82	yes	1.89E-02	yes	Down
TFEB	0.82	yes	3.97E-02	yes	Down
HSPA1A	0.82	yes	2.71E-02	yes	Down
FLAD1	0.82	yes	4.18E-02	yes	Down
JDP2	0.82	yes	2.81E-02	yes	Down
THOC5	0.82	yes	3.43E-02	yes	Down
PELO	0.82	yes	4.60E-02	yes	Down
PILRA	0.82	yes	4.78E-02	yes	Down
MTX1	0.82	yes	4.89E-02	yes	Down
PDLIM7	0.82	yes	2.37E-02	yes	Down
ELL	0.81	yes	3.29E-02	yes	Down
PGD	0.81	yes	3.57E-02	yes	Down
DUSP2	0.81	yes	4.54E-02	yes	Down
APBB1IP	0.81	yes	1.30E-02	yes	Down
GABARAP	0.81	yes	2.03E-02	yes	Down
TNFRSF1B	0.81	yes	3.15E-02	yes	Down
VNN2	0.81	yes	3.39E-02	yes	Down
PACSIN2	0.81	yes	4.10E-02	yes	Down
DOK3	0.81	yes	2.33E-02	yes	Down
ZNF324	0.81	yes	3.96E-02	yes	Down
PPCDC	0.81	yes	4.66E-02	yes	Down
FLOT1	0.81	yes	2.98E-02	yes	Down
GRK6	0.81	yes	2.79E-02	yes	Down
JPT1	0.81	yes	1.47E-02	yes	Down
MMP9	0.81	yes	2.90E-02	yes	Down
ACTN1	0.81	yes	4.31E-02	yes	Down
GLB1	0.81	yes	3.88E-02	yes	Down

PSME3	0.81	yes	2.44E-02	yes	Down
DAPK2	0.81	yes	1.27E-02	yes	Down
ST6GALNAC2	0.81	yes	4.14E-02	yes	Down
PINK1	0.81	yes	2.06E-02	yes	Down
B3GNT8	0.81	yes	4.52E-02	yes	Down
RPS6KA1	0.81	yes	3.26E-02	yes	Down
NAIF1	0.81	yes	4.03E-02	yes	Down
VIM	0.81	yes	3.27E-02	yes	Down
DNAJB9	0.81	yes	4.90E-02	yes	Down
NCF4	0.81	yes	1.31E-02	yes	Down
TPD52L2	0.81	yes	1.13E-02	yes	Down
LILRB3	0.81	yes	3.94E-02	yes	Down
NATD1	0.81	yes	2.66E-02	yes	Down
CCN3	0.81	yes	3.64E-02	yes	Down
OAZ2	0.81	yes	1.38E-02	yes	Down
MAP2K3	0.81	yes	2.96E-02	yes	Down
RNF135	0.81	yes	2.06E-02	yes	Down
STARD10	0.81	yes	2.46E-02	yes	Down
ZER1	0.81	yes	3.99E-02	yes	Down
TMEM120A	0.81	yes	3.19E-02	yes	Down
MLF2	0.81	yes	2.61E-02	yes	Down
SLC12A9	0.81	yes	3.53E-02	yes	Down
PPP4C	0.81	yes	1.53E-02	yes	Down
YPEL5	0.81	yes	3.54E-02	yes	Down
SHKBP1	0.81	yes	4.19E-02	yes	Down
LRP10	0.80	yes	4.32E-02	yes	Down
KDM4B	0.80	yes	4.22E-02	yes	Down
SAMD1	0.80	yes	4.59E-02	yes	Down
TGFA	0.80	yes	2.29E-02	yes	Down
TFE3	0.80	yes	2.30E-02	yes	Down
RIN3	0.80	yes	2.12E-02	yes	Down
ST3GAL2	0.80	yes	3.74E-02	yes	Down
TPI1	0.80	yes	3.84E-02	yes	Down
MSRB1	0.80	yes	3.36E-02	yes	Down
CSF3R	0.80	yes	4.94E-02	yes	Down
LITAF	0.80	yes	1.18E-02	yes	Down
PGM1	0.80	yes	2.72E-02	yes	Down
MAPK3	0.80	yes	1.38E-02	yes	Down
GNG7	0.80	yes	3.93E-02	yes	Down
CXCR2	0.80	yes	2.36E-02	yes	Down
XPO6	0.80	yes	2.66E-02	yes	Down
IFITM2	0.80	yes	1.04E-02	yes	Down
SLC39A1	0.80	yes	4.12E-02	yes	Down
PGAP6	0.80	yes	4.53E-02	yes	Down

CLTB	0.80	yes	2.24E-02	yes	Down
RPN1	0.80	yes	2.74E-02	yes	Down
PCIF1	0.80	yes	3.26E-02	yes	Down
RHOG	0.80	yes	3.04E-02	yes	Down
RTN3	0.80	yes	8.65E-03	yes	Down
ARAP3	0.80	yes	1.96E-02	yes	Down
FKBP1A	0.80	yes	1.54E-02	yes	Down
RASGRP4	0.80	yes	3.40E-02	yes	Down
PPT1	0.80	yes	7.37E-03	yes	Down
DXO	0.80	yes	1.68E-02	yes	Down
DNAJB12	0.80	yes	4.08E-02	yes	Down
VSIR	0.80	yes	4.29E-02	yes	Down
GGT1	0.80	yes	3.55E-02	yes	Down
PXN	0.80	yes	2.15E-02	yes	Down
DEDD2	0.80	yes	2.46E-02	yes	Down
MOB3A	0.80	yes	2.72E-02	yes	Down
IFNGR2	0.80	yes	2.12E-02	yes	Down
KLF11	0.80	yes	1.39E-02	yes	Down
CD79A	0.80	yes	4.74E-02	yes	Down
SLC15A4	0.80	yes	1.87E-02	yes	Down
LSP1	0.80	yes	2.03E-02	yes	Down
RABAC1	0.80	yes	1.34E-02	yes	Down
LRRC4	0.80	yes	3.39E-02	yes	Down
B3GNTL1	0.80	yes	3.71E-02	yes	Down
QPCT	0.80	yes	1.35E-02	yes	Down
CYTH4	0.80	yes	2.79E-02	yes	Down
NARF	0.80	yes	1.91E-02	yes	Down
RUBCNL	0.80	yes	6.23E-03	yes	Down
FCHO1	0.79	yes	2.27E-02	yes	Down
TREML2	0.79	yes	2.82E-02	yes	Down
LOC400499	0.79	yes	1.50E-02	yes	Down
PLOD1	0.79	yes	3.67E-02	yes	Down
RIPOR1	0.79	yes	4.03E-02	yes	Down
PLPPR2	0.79	yes	2.78E-02	yes	Down
GNAI2	0.79	yes	2.05E-02	yes	Down
PPIF	0.79	yes	2.25E-02	yes	Down
SIGLEC5	0.79	yes	5.45E-03	yes	Down
S100A9	0.79	yes	6.80E-03	yes	Down
SPI1	0.79	yes	1.15E-02	yes	Down
ZNF646	0.79	yes	1.57E-02	yes	Down
PRKACA	0.79	yes	2.42E-02	yes	Down
S100A11	0.79	yes	6.69E-03	yes	Down
MAST3	0.79	yes	3.74E-02	yes	Down
CCDC71L	0.79	yes	1.62E-02	yes	Down

NFE4	0.79	yes	2.72E-02	yes	Down
NOTCH1	0.79	yes	2.90E-02	yes	Down
CASS4	0.79	yes	1.02E-02	yes	Down
COTL1	0.79	yes	2.68E-02	yes	Down
TOM1	0.79	yes	1.08E-02	yes	Down
TALDO1	0.79	yes	2.47E-02	yes	Down
RHOB	0.79	yes	2.36E-02	yes	Down
GPR27	0.79	yes	2.89E-02	yes	Down
BAZ2B-AS1	0.79	yes	1.05E-02	yes	Down
FRAT1	0.79	yes	7.26E-03	yes	Down
NIBAN1	0.79	yes	2.63E-02	yes	Down
AQP9	0.79	yes	2.80E-02	yes	Down
NPTXR	0.79	yes	4.86E-02	yes	Down
USB1	0.79	yes	9.06E-03	yes	Down
TNFRSF1A	0.79	yes	3.43E-02	yes	Down
TUBB4B	0.79	yes	2.38E-02	yes	Down
BCL3	0.79	yes	2.95E-02	yes	Down
GPAT3	0.79	yes	9.40E-03	yes	Down
ZBTB7B	0.79	yes	1.53E-02	yes	Down
PHC2	0.79	yes	4.53E-02	yes	Down
RNF26	0.79	yes	4.47E-02	yes	Down
SLC43A2	0.79	yes	2.92E-02	yes	Down
YIPF1	0.79	yes	1.56E-02	yes	Down
BTNL8	0.79	yes	1.49E-02	yes	Down
ZDHHC4	0.79	yes	4.94E-02	yes	Down
PPP1R18	0.79	yes	1.23E-02	yes	Down
LETM2	0.79	yes	3.33E-02	yes	Down
ARL8A	0.79	yes	1.23E-02	yes	Down
LOC101928143	0.79	yes	1.10E-02	yes	Down
ANP32A-IT1	0.79	yes	4.53E-02	yes	Down
IRAG1	0.79	yes	1.22E-02	yes	Down
DHX34	0.79	yes	1.37E-02	yes	Down
SH2B2	0.79	yes	2.10E-02	yes	Down
ZFP36L1	0.79	yes	9.95E-03	yes	Down
PCBP1	0.78	yes	1.03E-02	yes	Down
NDE1	0.78	yes	1.12E-02	yes	Down
UBALD1	0.78	yes	3.17E-02	yes	Down
VASP	0.78	yes	7.69E-03	yes	Down
ALYREF	0.78	yes	1.94E-02	yes	Down
NEU1	0.78	yes	6.20E-03	yes	Down
TKT	0.78	yes	2.49E-02	yes	Down
NFE2	0.78	yes	5.76E-03	yes	Down
DNTTIP1	0.78	yes	8.38E-03	yes	Down
PPP4R1	0.78	yes	1.83E-02	yes	Down

CPVL	0.78	yes	2.90E-02	yes	Down
FAM214B	0.78	yes	2.99E-02	yes	Down
ZNF768	0.78	yes	2.95E-02	yes	Down
LILRA2	0.78	yes	1.13E-02	yes	Down
SEMA4A	0.78	yes	1.95E-02	yes	Down
GPSM3	0.78	yes	1.63E-02	yes	Down
CNN2	0.78	yes	1.00E-02	yes	Down
MBOAT7	0.78	yes	4.06E-02	yes	Down
LSR	0.78	yes	3.42E-02	yes	Down
ASGR2	0.78	yes	4.86E-02	yes	Down
PLD3	0.78	yes	3.97E-02	yes	Down
SLC11A1	0.78	yes	4.51E-02	yes	Down
UBE2M	0.78	yes	1.26E-02	yes	Down
RARA	0.78	yes	1.06E-02	yes	Down
CPPED1	0.78	yes	3.25E-02	yes	Down
ICAM3	0.78	yes	2.70E-02	yes	Down
FBXL12	0.78	yes	7.54E-03	yes	Down
ARMC5	0.78	yes	3.51E-02	yes	Down
XKR8	0.78	yes	3.10E-02	yes	Down
BRI3	0.78	yes	2.28E-03	yes	Down
TSEN34	0.78	yes	9.54E-03	yes	Down
ACP3	0.78	yes	3.52E-02	yes	Down
MANSC1	0.78	yes	5.85E-03	yes	Down
ECE1	0.78	yes	2.98E-02	yes	Down
SLC25A37	0.78	yes	1.20E-02	yes	Down
DDIT3	0.78	yes	2.70E-02	yes	Down
LRG1	0.78	yes	8.36E-03	yes	Down
FTL	0.78	yes	4.48E-03	yes	Down
NLRP12	0.78	yes	6.88E-03	yes	Down
RBM47	0.78	yes	1.40E-02	yes	Down
CBX4	0.78	yes	9.45E-03	yes	Down
HMOX1	0.78	yes	3.23E-02	yes	Down
AURKAIP1	0.78	yes	1.86E-02	yes	Down
ACSL1	0.78	yes	2.27E-02	yes	Down
UBE2R2	0.77	yes	4.21E-03	yes	Down
LSM10	0.77	yes	9.20E-03	yes	Down
FMNL1-AS1	0.77	yes	4.36E-02	yes	Down
HCAR2	0.77	yes	2.46E-02	yes	Down
ZNF668	0.77	yes	2.54E-02	yes	Down
CANT1	0.77	yes	9.38E-03	yes	Down
LIMK2	0.77	yes	3.42E-02	yes	Down
MMP25	0.77	yes	1.50E-02	yes	Down
MGAM	0.77	yes	2.95E-02	yes	Down
NINJ1	0.77	yes	1.27E-02	yes	Down

CDA	0.77	yes	3.27E-03	yes	Down
CHSY1	0.77	yes	4.85E-03	yes	Down
MKNK2	0.77	yes	2.37E-02	yes	Down
IL4R	0.77	yes	1.33E-02	yes	Down
GRAMD1A	0.77	yes	9.96E-03	yes	Down
CEACAM3	0.77	yes	3.52E-03	yes	Down
CA4	0.77	yes	1.50E-02	yes	Down
SCYL1	0.77	yes	9.89E-03	yes	Down
ATP6V0B	0.77	yes	2.59E-03	yes	Down
CKAP4	0.77	yes	1.93E-02	yes	Down
JUND	0.77	yes	2.65E-02	yes	Down
B9D2	0.77	yes	1.51E-02	yes	Down
CEBPD	0.77	yes	3.09E-02	yes	Down
DNAJB1	0.77	yes	6.20E-03	yes	Down
SIRPA	0.77	yes	2.26E-02	yes	Down
RGL4	0.77	yes	5.87E-03	yes	Down
PTP4A3	0.77	yes	1.50E-02	yes	Down
ZYX	0.77	yes	1.84E-02	yes	Down
FAM53C	0.77	yes	1.33E-02	yes	Down
SGK1	0.77	yes	1.99E-02	yes	Down
GNB2	0.77	yes	1.36E-02	yes	Down
FFAR2	0.77	yes	4.55E-03	yes	Down
AATK	0.77	yes	7.89E-03	yes	Down
LILRA5	0.77	yes	1.31E-02	yes	Down
FAM157A	0.77	yes	2.86E-02	yes	Down
S100A12	0.77	yes	3.91E-03	yes	Down
LOC105371934	0.77	yes	2.66E-02	yes	Down
BAIAP2	0.77	yes	4.71E-02	yes	Down
TMEM141	0.77	yes	1.56E-02	yes	Down
FCER1A	0.77	yes	3.96E-02	yes	Down
ECSIT	0.77	yes	2.64E-02	yes	Down
BCL6	0.77	yes	1.79E-02	yes	Down
LOC105374102	0.76	yes	3.80E-02	yes	Down
ERF	0.76	yes	3.07E-02	yes	Down
LSM2	0.76	yes	3.73E-02	yes	Down
MCEMP1	0.76	yes	1.40E-02	yes	Down
TMEM216	0.76	yes	2.28E-02	yes	Down
HSPC102	0.76	yes	5.46E-03	yes	Down
LOC105369161	0.76	yes	1.45E-02	yes	Down
IL1B	0.76	yes	3.27E-02	yes	Down
PADI2	0.76	yes	6.79E-03	yes	Down
FUT7	0.76	yes	1.28E-02	yes	Down
CXCR4	0.76	yes	7.17E-04	yes	Down
TLR5	0.76	yes	4.04E-03	yes	Down

ADGRE3	0.76	yes	7.29E-03	yes	Down
UBALD2	0.76	yes	2.21E-03	yes	Down
LOC254896	0.76	yes	4.09E-02	yes	Down
LPCAT3	0.76	yes	7.64E-03	yes	Down
ANPEP	0.76	yes	7.30E-03	yes	Down
CYP27A1	0.76	yes	3.02E-02	yes	Down
ETS2	0.76	yes	4.21E-03	yes	Down
TP53I11	0.76	yes	1.32E-02	yes	Down
PLIN3	0.76	yes	2.29E-03	yes	Down
PIM3	0.76	yes	1.62E-02	yes	Down
LRRC25	0.76	yes	8.08E-03	yes	Down
FARS2	0.76	yes	3.05E-02	yes	Down
CEBPB	0.76	yes	3.60E-03	yes	Down
PANX2	0.76	yes	1.83E-02	yes	Down
SPATA2	0.76	yes	9.81E-03	yes	Down
FPR1	0.75	yes	4.50E-03	yes	Down
RPH3A	0.75	yes	4.82E-02	yes	Down
EXT1	0.75	yes	1.44E-02	yes	Down
ZNF629	0.75	yes	2.65E-02	yes	Down
TECPR2	0.75	yes	6.50E-03	yes	Down
S100P	0.75	yes	2.38E-03	yes	Down
SCN1B	0.75	yes	2.20E-02	yes	Down
METRNL	0.75	yes	7.55E-03	yes	Down
KRT23	0.75	yes	2.78E-03	yes	Down
NR4A1	0.75	yes	2.71E-02	yes	Down
IMPA2	0.75	yes	2.05E-02	yes	Down
CXCR1	0.75	yes	1.91E-03	yes	Down
SLC15A3	0.75	yes	3.21E-03	yes	Down
LINC00963	0.75	yes	8.31E-03	yes	Down
FTH1	0.75	yes	1.64E-03	yes	Down
NECAB2	0.75	yes	2.76E-02	yes	Down
RGS2	0.75	yes	8.73E-03	yes	Down
TMEM88	0.75	yes	1.76E-02	yes	Down
LOC105375785	0.75	yes	1.46E-02	yes	Down
JMJD6	0.75	yes	1.06E-03	yes	Down
YRDC	0.75	yes	3.83E-03	yes	Down
HCK	0.75	yes	8.90E-03	yes	Down
MYO7B	0.75	yes	4.63E-02	yes	Down
NANS	0.75	yes	1.01E-02	yes	Down
GSEC	0.75	yes	3.03E-02	yes	Down
TMEM45B	0.74	yes	1.02E-02	yes	Down
CD14	0.74	yes	5.83E-03	yes	Down
ZDHHC18	0.74	yes	2.24E-03	yes	Down
CPA3	0.74	yes	4.43E-02	yes	Down

C11orf49	0.74	yes	4.24E-02	yes	Down
FRAT2	0.74	yes	1.90E-04	yes	Down
IER3	0.74	yes	3.64E-03	yes	Down
NQO2	0.74	yes	3.65E-03	yes	Down
LOC100507507	0.74	yes	1.33E-02	yes	Down
PLAUR	0.74	yes	1.05E-02	yes	Down
PLK3	0.74	yes	2.12E-02	yes	Down
PLB1	0.74	yes	6.85E-03	yes	Down
CPNE2	0.74	yes	1.17E-02	yes	Down
SOCS3	0.74	yes	3.30E-02	yes	Down
SERTAD1	0.74	yes	1.81E-02	yes	Down
C5AR1	0.74	yes	2.36E-03	yes	Down
LOC107984521	0.74	yes	1.57E-02	yes	Down
EIF1	0.74	yes	1.17E-03	yes	Down
ITPRIP	0.74	yes	2.86E-03	yes	Down
FAM174A	0.74	yes	3.73E-03	yes	Down
ZNF467	0.74	yes	2.07E-02	yes	Down
NECTIN1	0.74	yes	1.73E-02	yes	Down
NFKBIZ	0.74	yes	4.57E-03	yes	Down
CDC42EP2	0.74	yes	3.53E-02	yes	Down
CADM4	0.74	yes	2.69E-02	yes	Down
FBRS	0.73	yes	1.65E-03	yes	Down
TAGLN2	0.73	yes	1.40E-02	yes	Down
KDM6B	0.73	yes	2.22E-03	yes	Down
GLUL	0.73	yes	8.10E-03	yes	Down
SLC22A4	0.73	yes	4.13E-03	yes	Down
R3HDM4	0.73	yes	1.48E-03	yes	Down
ZNF784	0.73	yes	2.78E-02	yes	Down
DYSF	0.73	yes	6.52E-03	yes	Down
MARCKS	0.73	yes	9.02E-05	yes	Down
SLC2A3	0.73	yes	4.02E-04	yes	Down
PFKFB3	0.73	yes	4.82E-04	yes	Down
PHOSPHO1	0.73	yes	1.75E-02	yes	Down
GLT1D1	0.73	yes	2.16E-03	yes	Down
TNFRSF10C	0.73	yes	1.24E-03	yes	Down
TESC	0.73	yes	2.10E-02	yes	Down
TNFRSF8	0.73	yes	6.41E-03	yes	Down
DDIT4	0.73	yes	1.70E-02	yes	Down
MAPRE3	0.73	yes	3.62E-02	yes	Down
FLOT2	0.73	yes	9.73E-04	yes	Down
CHI3L1	0.73	yes	4.82E-03	yes	Down
KLF16	0.73	yes	1.11E-03	yes	Down
TTLL1	0.73	yes	2.75E-02	yes	Down
CEBPB-AS1	0.73	yes	1.26E-02	yes	Down

ANXA3	0.73	yes	4.53E-03	yes	Down
JUN	0.72	yes	7.34E-03	yes	Down
NR4A2	0.72	yes	2.28E-02	yes	Down
CD83	0.72	yes	5.11E-03	yes	Down
LGALS2	0.72	yes	1.67E-03	yes	Down
ASF1B	0.72	yes	8.10E-03	yes	Down
GPX3	0.72	yes	3.02E-02	yes	Down
TLE3	0.72	yes	1.16E-03	yes	Down
TUBA1A	0.72	yes	4.88E-03	yes	Down
SERPINA1	0.72	yes	2.99E-03	yes	Down
CFAP45	0.72	yes	8.35E-03	yes	Down
LOC107986939	0.72	yes	4.25E-02	yes	Down
LOC107984917	0.72	yes	4.14E-02	yes	Down
XPC-AS1	0.71	yes	9.24E-03	yes	Down
SLC29A3	0.71	yes	4.31E-02	yes	Down
MIDN	0.71	yes	2.78E-03	yes	Down
DHRS13	0.71	yes	1.02E-03	yes	Down
ZFAND2A	0.71	yes	5.33E-04	yes	Down
ATP2B1-AS1	0.71	yes	5.39E-04	yes	Down
LOC105377067	0.71	yes	9.49E-03	yes	Down
NFKBIA	0.70	yes	2.01E-03	yes	Down
WLS	0.70	yes	7.25E-05	yes	Down
LOC399900	0.70	yes	1.60E-02	yes	Down
H2BC21	0.70	yes	1.10E-03	yes	Down
COL5A3	0.70	yes	4.12E-02	yes	Down
AMPD2	0.70	yes	1.52E-02	yes	Down
SLPI	0.70	yes	1.20E-02	yes	Down
SLC22A1	0.70	yes	1.41E-02	yes	Down
LOC112268067	0.70	yes	8.36E-04	yes	Down
ELAPOR1	0.70	yes	1.13E-03	yes	Down
ARHGEF40	0.70	yes	1.06E-02	yes	Down
LOC105379321	0.70	yes	4.80E-02	yes	Down
ZNF608	0.70	yes	2.38E-02	yes	Down
HRH2	0.70	yes	1.09E-03	yes	Down
LOC105372323	0.70	yes	4.17E-02	yes	Down
H2AC19	0.70	yes	3.94E-02	yes	Down
ADGRE2	0.70	yes	4.79E-04	yes	Down
ZFP36	0.70	yes	5.74E-03	yes	Down
PWP2	0.70	yes	3.74E-02	yes	Down
DGAT2	0.69	yes	7.66E-04	yes	Down
SRF	0.69	yes	7.25E-04	yes	Down
PKIG	0.69	yes	1.53E-02	yes	Down
CCDC153	0.69	yes	4.20E-02	yes	Down
THBD	0.69	yes	9.38E-04	yes	Down

RRP12	0.69	yes	8.93E-04	yes	Down
TRIM8	0.69	yes	2.76E-04	yes	Down
MAK	0.69	yes	5.22E-03	yes	Down
TRIB1	0.69	yes	1.29E-03	yes	Down
NFIL3	0.68	yes	6.19E-04	yes	Down
CFAP92	0.68	yes	3.76E-03	yes	Down
ADGRG3	0.68	yes	1.21E-03	yes	Down
MYADM	0.68	yes	1.98E-03	yes	Down
LOC105371088	0.68	yes	1.21E-02	yes	Down
TPPP3	0.68	yes	1.81E-02	yes	Down
ZNF394	0.68	yes	4.53E-05	yes	Down
PDK4	0.68	yes	9.16E-03	yes	Down
LOC107986153	0.68	yes	1.26E-02	yes	Down
PLK2	0.67	yes	2.15E-02	yes	Down
BASP1	0.67	yes	1.70E-05	yes	Down
KAZN	0.67	yes	1.09E-03	yes	Down
ST3GAL4	0.67	yes	1.20E-04	yes	Down
PPP1R15A	0.67	yes	9.36E-04	yes	Down
PGLYRP1	0.67	yes	6.77E-03	yes	Down
SDC2	0.66	yes	7.92E-03	yes	Down
FOS	0.66	yes	1.72E-03	yes	Down
FCAR	0.66	yes	4.80E-06	yes	Down
LINC01506	0.66	yes	7.93E-05	yes	Down
RAB36	0.66	yes	2.93E-02	yes	Down
SAT1	0.66	yes	6.02E-04	yes	Down
BTG2	0.66	yes	1.14E-04	yes	Down
TGM3	0.66	yes	5.52E-03	yes	Down
TNFRSF21	0.66	yes	4.64E-02	yes	Down
MAFF	0.66	yes	6.52E-03	yes	Down
NLRP6	0.66	yes	1.48E-05	yes	Down
LINC00528	0.66	yes	8.75E-05	yes	Down
LOC101928344	0.66	yes	4.12E-03	yes	Down
ELF3	0.66	yes	3.76E-02	yes	Down
CYSTM1	0.66	yes	5.89E-04	yes	Down
HP	0.66	yes	2.29E-02	yes	Down
UBTD1	0.65	yes	3.48E-03	yes	Down
LOC107987250	0.65	yes	9.47E-04	yes	Down
NRG1	0.65	yes	4.11E-03	yes	Down
BTG2-DT	0.65	yes	3.36E-02	yes	Down
LOC107984583	0.65	yes	3.64E-03	yes	Down
MIR22HG	0.65	yes	9.04E-04	yes	Down
LOC101928361	0.65	yes	2.12E-02	yes	Down
LOC107987081	0.65	yes	1.95E-02	yes	Down
LOC102724608	0.65	yes	7.75E-04	yes	Down

MIR23AHG	0.65	yes	3.58E-03	yes	Down
LOC102723996	0.64	yes	1.81E-02	yes	Down
CYB5D1	0.64	yes	2.01E-04	yes	Down
LOC107986929	0.64	yes	1.01E-02	yes	Down
TREM1	0.64	yes	2.01E-04	yes	Down
PGM5	0.64	yes	3.84E-02	yes	Down
H2AC18	0.64	yes	2.48E-03	yes	Down
CD1A	0.64	yes	4.81E-02	yes	Down
CATIP	0.64	yes	3.22E-03	yes	Down
JUNB	0.64	yes	8.90E-04	yes	Down
LINC01094	0.63	yes	3.30E-03	yes	Down
CNTNAP3	0.63	yes	2.35E-03	yes	Down
LOC101929331	0.63	yes	1.26E-04	yes	Down
LOC107984334	0.63	yes	3.37E-03	yes	Down
IER2	0.63	yes	8.23E-05	yes	Down
TNFRSF9	0.63	yes	9.19E-04	yes	Down
SIGLEC12	0.63	yes	3.01E-02	yes	Down
CCNJL	0.63	yes	9.76E-06	yes	Down
PPP1R35-AS1	0.63	yes	7.22E-03	yes	Down
ZNF628	0.62	yes	2.88E-03	yes	Down
PROK2	0.62	yes	1.03E-06	yes	Down
OTX1	0.62	yes	2.07E-02	yes	Down
SMPDL3B	0.62	yes	1.31E-02	yes	Down
DUSP1	0.61	yes	5.99E-06	yes	Down
LOC105372578	0.61	yes	2.34E-02	yes	Down
LOC112268100	0.61	yes	3.14E-03	yes	Down
DOK4	0.60	yes	6.54E-04	yes	Down
LOC105377016	0.60	yes	5.61E-04	yes	Down
RPP25	0.60	yes	3.57E-02	yes	Down
ADM	0.60	yes	3.56E-07	yes	Down
LINC01353	0.60	yes	1.19E-03	yes	Down
FFAR3	0.60	yes	3.20E-02	yes	Down
RAB3A	0.59	yes	4.33E-02	yes	Down
LOC107987234	0.59	yes	2.24E-02	yes	Down
ARHGAP26-IT1	0.59	yes	4.97E-02	yes	Down
ORM1	0.59	yes	1.58E-02	yes	Down
FOLR2	0.58	yes	3.27E-02	yes	Down
SFN	0.58	yes	1.15E-03	yes	Down
LOC101927954	0.58	yes	4.42E-02	yes	Down
MXRA8	0.58	yes	1.04E-02	yes	Down
TP53INP2	0.57	yes	8.82E-06	yes	Down
LOC105370877	0.57	yes	1.31E-03	yes	Down
NTN4	0.57	yes	1.73E-02	yes	Down
CSRNP1	0.57	yes	8.59E-06	yes	Down

LOC105373759	0.57	yes	2.02E-02	yes	Down
BIK	0.56	yes	2.75E-02	yes	Down
LOC114224	0.56	yes	2.16E-02	yes	Down
LOC105378620	0.56	yes	9.23E-06	yes	Down
RFX2	0.56	yes	3.16E-04	yes	Down
CXCL1	0.56	yes	9.28E-08	yes	Down
ACVRL1	0.56	yes	3.66E-02	yes	Down
UBE2C	0.56	yes	1.27E-04	yes	Down
PTGS2	0.56	yes	5.51E-06	yes	Down
ALPL	0.55	yes	3.39E-06	yes	Down
MYBPH	0.55	yes	3.66E-02	yes	Down
LRRC46	0.55	yes	1.89E-02	yes	Down
ENTPD2	0.55	yes	2.02E-02	yes	Down
LOC107986363	0.55	yes	3.47E-02	yes	Down
EGR2	0.55	yes	1.07E-06	yes	Down
LINC02596	0.54	yes	5.99E-03	yes	Down
KL	0.54	yes	4.89E-02	yes	Down
ITGB4	0.54	yes	1.02E-02	yes	Down
LOC107984529	0.54	yes	2.12E-04	yes	Down
LOC105369180	0.54	yes	1.97E-04	yes	Down
DNAJB5	0.53	yes	1.30E-03	yes	Down
GALNT14	0.53	yes	2.46E-04	yes	Down
CXCL8	0.52	yes	8.41E-03	yes	Down
PACERR	0.52	yes	2.10E-02	yes	Down
LINC01220	0.52	yes	2.16E-02	yes	Down
LOC112268131	0.52	yes	6.82E-03	yes	Down
CD177	0.51	yes	2.51E-06	yes	Down
LOC105379322	0.51	yes	5.06E-03	yes	Down
C17orf107	0.51	yes	5.19E-04	yes	Down
LOC105376568	0.50	yes	1.71E-05	yes	Down
LOC112268195	0.49	yes	6.30E-03	yes	Down
LINC01888	0.49	yes	8.25E-04	yes	Down
LOC105376504	0.49	yes	5.95E-03	yes	Down
HBEGF	0.48	yes	6.72E-06	yes	Down
FSCN3	0.48	yes	2.91E-03	yes	Down
FOSL1	0.47	yes	9.13E-03	yes	Down
EGR3	0.47	yes	1.06E-07	yes	Down
MAP3K15	0.47	yes	6.09E-04	yes	Down
MPP2	0.46	yes	3.36E-02	yes	Down
H3C1	0.46	yes	1.90E-02	yes	Down
LOC105377730	0.46	yes	1.05E-09	yes	Down
MAPK8IP2	0.46	yes	4.08E-02	yes	Down
INSC	0.46	yes	8.37E-03	yes	Down
EFNA1	0.45	yes	3.66E-03	yes	Down

EGR1	0.45	yes	3.78E-08	yes	Down
LOC105372709	0.45	yes	1.52E-02	yes	Down
ADPGK-AS1	0.45	yes	3.01E-03	yes	Down
OSM	0.44	yes	7.92E-10	yes	Down
LINC00664	0.44	yes	1.89E-03	yes	Down
LOC105372421	0.41	yes	3.18E-02	yes	Down
ENHO	0.41	yes	2.56E-02	yes	Down
LOC105375554	0.40	yes	4.79E-02	yes	Down
LOC107986364	0.40	yes	1.10E-03	yes	Down
LOC105372681	0.40	yes	2.95E-02	yes	Down
BABAM2-AS1	0.39	yes	1.08E-10	yes	Down
TBC1D3	0.39	yes	2.05E-02	yes	Down
ARAP1-AS2	0.38	yes	4.93E-03	yes	Down
LOC107986926	0.37	yes	3.83E-03	yes	Down
CFAP141	0.37	yes	3.08E-02	yes	Down
POU5F1	0.37	yes	8.33E-04	yes	Down
G0S2	0.36	yes	1.84E-22	yes	Down
LOC101927949	0.36	yes	1.42E-03	yes	Down
MADCAM1	0.36	yes	4.27E-03	yes	Down
LOC112268070	0.36	yes	1.63E-02	yes	Down
RNF222	0.35	yes	1.03E-03	yes	Down
TNRC6B-DT	0.34	yes	1.13E-02	yes	Down
B3GNT4	0.34	yes	4.67E-02	yes	Down
IQCD	0.33	yes	4.61E-02	yes	Down
SDS	0.32	yes	4.86E-02	yes	Down
RGS2-AS1	0.32	yes	2.65E-02	yes	Down
ECRG4	0.32	yes	2.25E-02	yes	Down
LOC105374264	0.31	yes	1.45E-02	yes	Down
CCDC116	0.31	yes	2.71E-02	yes	Down
FAM27E4	0.30	yes	2.75E-02	yes	Down
OR6K3	0.30	yes	3.64E-02	yes	Down
PEF1-AS1	0.29	yes	4.48E-02	yes	Down
LOC102724594	0.27	yes	2.84E-03	yes	Down
PRSS16	0.26	yes	2.97E-03	yes	Down
LOC101927164	0.23	yes	2.36E-02	yes	Down
SOX6	0.22	yes	4.12E-02	yes	Down
PLAAT2	0.21	yes	1.34E-02	yes	Down
LOC105375426	0.19	yes	2.81E-02	yes	Down
LOC105374906	0.19	yes	7.61E-03	yes	Down
FAM222A	0.17	yes	6.38E-04	yes	Down
EPCAM	0.17	yes	1.16E-02	yes	Down
COL1A1	0.16	yes	3.56E-02	yes	Down
GPR17	0.15	yes	2.56E-02	yes	Down
FAM223A	0.15	yes	4.38E-02	yes	Down

JAKMIP3-AS1	0.08	yes	4.63E-02	yes	Down
PRSS3	0.05	yes	1.72E-03	yes	Down
