# Supplementary material for: Transcriptomic analysis reveals the key role of inflammatory and immune signaling in the anti-perimenopausal depression effects of Bushen Shugan Huayu decoction
Source: Front Psychiatry. 2025 Sep 26;16:1629900. doi: 10.3389/fpsyt.2025.1629900 (PMC12512047; doi:10.3389/fpsyt.2025.1629900)
Supplement: Supplementary file 3 [file Table3.pdf]

**Table S3** GO enrichment analysis ( *p*-value < 0.05 ). 492 GO terms are identified using the filter $p < 0.05$. The category, the term, the number of DEGs, *p*-value, and the enrichment score (ES) are described in the table.

# Table S3 GO enrichment analysis ( *p*-value < 0.05 )

| Category | ID | Term | Number | *p*-value | ES |
|---|---|---|---|---|---|
| molecular function | GO:0005515 | protein binding | 858 | 2.01E-08 | 1.11 |
| cellular component | GO:0005634 | vnucleus | 424 | 6.87E-08 | 1.24 |
| molecular function | GO:0000978 | RNA polymerase II cis-regulatory region sequence-specific DNA binding | 114 | 2.25E-07 | 1.61 |
| cellular component | GO:0005829 | cytosol | 408 | 5.36E-07 | 1.22 |
| cellular component | GO:0034451 | centriolar satellite | 23 | 7.45E-07 | 3.16 |
| cellular component | GO:1904724 | tertiary granule lumen | 15 | 7.48E-07 | 4.42 |
| biological process | GO:0006954 | inflammatory response | 50 | 9.12E-07 | 2.06 |
| biological process | GO:0050729 | positive regulation of inflammatory response | 20 | 1.55E-06 | 3.34 |
| biological process | GO:0090316 | positive regulation of intracellular protein transport | 9 | 3.00E-06 | 6.63 |
| biological process | GO:1905515 | non-motile cilium assembly | 14 | 6.73E-06 | 3.98 |
| biological process | GO:0140467 | integrated stress response signaling | 9 | 7.04E-06 | 6.08 |
| cellular component | GO:0005654 | nucleoplasm | 299 | 1.03E-05 | 1.24 |
| cellular component | GO:0005886 | plasma membrane | 361 | 1.73E-05 | 1.20 |
| biological process | GO:0045944 | positive regulation of transcription by RNA polymerase II | 108 | 1.77E-05 | 1.49 |
| cellular component | GO:0030667 | secretory granule membrane | 18 | 2.03E-05 | 3.04 |
| cellular component | GO:0005737 | cytoplasm | 367 | 2.83E-05 | 1.20 |
| cellular component | GO:0035976 | transcription factor AP-1 complex | 4 | 6.85E-05 | 12.97 |
| cellular component | GO:0001726 | ruffle | 17 | 8.34E-05 | 2.84 |
| cellular component | GO:0035580 | specific granule lumen | 13 | 8.55E-05 | 3.40 |
| biological process | GO:0030593 | neutrophil chemotaxis | 15 | 9.76E-05 | 3.04 |

| | | | | | |
|---|---|---|---|---|---|
| molecular function | GO:0001228 | DNA-binding transcription activator activity, RNA polymerase II-specific | 50 | 1.03E-04 | 1.74 |
| cellular component | GO:0070062 | extracellular exosome | 169 | 1.23E-04 | 1.31 |
| cellular component | GO:0005813 | centrosome | 56 | 1.29E-04 | 1.66 |
| cellular component | GO:0005794 | Golgi apparatus | 92 | 1.30E-04 | 1.47 |
| molecular function | GO:0003677 | DNA binding | 87 | 1.35E-04 | 1.48 |
| biological process | GO:0006974 | DNA damage response | 33 | 1.47E-04 | 1.97 |
| biological process | GO:1903140 | regulation of establishment of endothelial barrier | 5 | 1.72E-04 | 8.11 |
| biological process | GO:0044344 | cellular response to fibroblast growth factor stimulus | 8 | 2.09E-04 | 4.63 |
| biological process | GO:0070266 | necroptotic process | 6 | 2.55E-04 | 6.08 |
| biological process | GO:0019221 | cytokine-mediated signaling pathway | 20 | 3.42E-04 | 2.33 |
| biological process | GO:0006955 | immune response | 34 | 3.80E-04 | 1.86 |
| cellular component | GO:0005856 | cytoskeleton | 45 | 4.52E-04 | 1.68 |
| cellular component | GO:0015629 | actin cytoskeleton | 30 | 4.55E-04 | 1.92 |
| biological process | GO:0006357 | regulation of transcription by RNA polymerase II | 121 | 4.70E-04 | 1.34 |
| molecular function | GO:0035259 | nuclear glucocorticoid receptor binding | 5 | 4.87E-04 | 6.75 |
| biological process | GO:0032496 | response to lipopolysaccharide | 19 | 5.21E-04 | 2.32 |
| molecular function | GO:0046872 | metal ion binding | 181 | 6.87E-04 | 1.25 |
| cellular component | GO:0031252 | cell leading edge | 10 | 6.93E-04 | 3.31 |
| molecular function | GO:0005524 | ATP binding | 119 | 9.72E-04 | 1.32 |
| cellular component | GO:0101003 | ficolin-1-rich granule membrane | 11 | 9.85E-04 | 2.97 |
| cellular component | GO:0035579 | specific granule membrane | 14 | 1.18E-03 | 2.52 |
| cellular component | GO:0090575 | RNA polymerase II transcription regulator complex | 17 | 1.21E-03 | 2.28 |
| biological process | GO:0001817 | regulation of cytokine production | 11 | 1.30E-03 | 2.88 |
| biological process | GO:0071243 | cellular response to arsenic-containing substance | 4 | 1.41E-03 | 7.20 |
| biological process | GO:0032757 | positive regulation of interleukin-8 production | 11 | 1.49E-03 | 2.83 |
| molecular function | GO:0003924 | GTPase activity | 34 | 1.58E-03 | 1.71 |

| | | | | | |
|---|---|---|---|---|---|
| biological process | GO:2000145 | regulation of cell motility | 5 | 1.58E-03 | 5.40 |
| biological process | GO:0008380 | RNA splicing | 22 | 1.68E-03 | 1.98 |
| biological process | GO:0006366 | transcription by RNA polymerase II | 26 | 1.74E-03 | 1.86 |
| cellular component | GO:0016607 | nuclear speck | 41 | 1.99E-03 | 1.60 |
| biological process | GO:0042713 | sperm ejaculation | 3 | 2.13E-03 | 9.73 |
| biological process | GO:1990743 | protein sialylation | 3 | 2.13E-03 | 9.73 |
| molecular function | GO:0000981 | DNA-binding transcription factor activity, RNA polymerase II-specific | 96 | 2.16E-03 | 1.33 |
| biological process | GO:0051298 | centrosome duplication | 5 | 2.18E-03 | 5.07 |
| biological process | GO:0006909 | phagocytosis | 11 | 2.20E-03 | 2.70 |
| biological process | GO:0051493 | regulation of cytoskeleton organization | 6 | 2.21E-03 | 4.23 |
| biological process | GO:0000722 | telomere maintenance via recombination | 4 | 2.24E-03 | 6.48 |
| molecular function | GO:0050786 | RAGE receptor binding | 4 | 2.24E-03 | 6.48 |
| molecular function | GO:0051880 | G-quadruplex DNA binding | 4 | 2.24E-03 | 6.48 |
| biological process | GO:0090398 | cellular senescence | 10 | 2.33E-03 | 2.84 |
| biological process | GO:0006397 | mRNA processing | 22 | 2.38E-03 | 1.93 |
| molecular function | GO:0004386 | helicase activity | 10 | 2.66E-03 | 2.79 |
| biological process | GO:0008625 | extrinsic apoptotic signaling pathway via death domain receptors | 8 | 2.68E-03 | 3.24 |
| cellular component | GO:0005765 | lysosomal membrane | 37 | 2.74E-03 | 1.62 |
| biological process | GO:0042770 | signal transduction in response to DNA damage | 5 | 2.93E-03 | 4.77 |
| biological process | GO:0045638 | negative regulation of myeloid cell differentiation | 5 | 2.93E-03 | 4.77 |
| biological process | GO:0006968 | cellular defense response | 9 | 3.17E-03 | 2.92 |
| biological process | GO:0035020 | regulation of Rac protein signal transduction | 4 | 3.35E-03 | 5.89 |
| biological process | GO:1901731 | positive regulation of platelet aggregation | 4 | 3.35E-03 | 5.89 |
| cellular component | GO:0035869 | ciliary transition zone | 7 | 3.46E-03 | 3.44 |
| molecular function | GO:0140658 | ATP-dependent chromatin remodeler activity | 7 | 3.46E-03 | 3.44 |
| molecular function | GO:0034237 | protein kinase A regulatory subunit binding | 6 | 3.49E-03 | 3.89 |

| | | | | | |
|---|---|---|---|---|---|
| biological process | GO:0090200 | positive regulation of release of cytochrome c from mitochondria | 6 | 3.49E-03 | 3.89 |
| biological process | GO:0045444 | fat cell differentiation | 12 | 3.53E-03 | 2.43 |
| cellular component | GO:0000781 | chromosome, telomeric region | 20 | 3.62E-03 | 1.93 |
| biological process | GO:0071364 | cellular response to epidermal growth factor stimulus | 8 | 3.69E-03 | 3.09 |
| biological process | GO:2000114 | regulation of establishment of cell polarity | 5 | 3.86E-03 | 4.50 |
| biological process | GO:0009052 | pentose-phosphate shunt, non-oxidative branch | 3 | 4.07E-03 | 8.11 |
| biological process | GO:0070434 | positive regulation of nucleotide-binding oligomerization domain containing 2 signaling pathway | 3 | 4.07E-03 | 8.11 |
| biological process | GO:0097398 | cellular response to interleukin-17 | 3 | 4.07E-03 | 8.11 |
| biological process | GO:1990776 | response to angiotensin | 3 | 4.07E-03 | 8.11 |
| biological process | GO:2000144 | positive regulation of DNA-templated transcription initiation | 3 | 4.07E-03 | 8.11 |
| biological process | GO:0045742 | positive regulation of epidermal growth factor receptor signaling pathway | 6 | 4.31E-03 | 3.74 |
| cellular component | GO:0051233 | spindle midzone | 6 | 4.31E-03 | 3.74 |
| biological process | GO:0006935 | chemotaxis | 15 | 4.34E-03 | 2.13 |
| biological process | GO:0007249 | canonical NF-kappaB signal transduction | 10 | 4.39E-03 | 2.61 |
| biological process | GO:0071260 | cellular response to mechanical stimulus | 11 | 4.43E-03 | 2.48 |
| cellular component | GO:0005874 | microtubule | 30 | 4.74E-03 | 1.65 |
| biological process | GO:0006486 | protein glycosylation | 9 | 4.76E-03 | 2.75 |
| biological process | GO:0016075 | rRNA catabolic process | 4 | 4.78E-03 | 5.40 |
| biological process | GO:0034976 | response to endoplasmic reticulum stress | 12 | 4.79E-03 | 2.34 |
| biological process | GO:0009611 | response to wounding | 10 | 4.94E-03 | 2.57 |
| biological process | GO:0031638 | zymogen activation | 5 | 4.98E-03 | 4.27 |
| cellular component | GO:0005925 | focal adhesion | 40 | 5.01E-03 | 1.53 |
| biological process | GO:0007166 | cell surface receptor signaling pathway | 25 | 5.02E-03 | 1.74 |
| molecular function | GO:0106310 | protein serine kinase activity | 35 | 5.24E-03 | 1.58 |
| biological process | GO:0042832 | defense response to protozoan | 6 | 5.25E-03 | 3.60 |

| | | | | | |
|---|---|---|---|---|---|
| biological process | GO:0045954 | positive regulation of natural killer cell mediated cytotoxicity | 6 | 5.25E-03 | 3.60 |
| cellular component | GO:0015630 | microtubule cytoskeleton | 21 | 5.43E-03 | 1.83 |
| cellular component | GO:0005901 | caveola | 11 | 5.47E-03 | 2.41 |
| biological process | GO:0006468 | protein phosphorylation | 37 | 5.71E-03 | 1.55 |
| molecular function | GO:0051015 | actin filament binding | 23 | 5.73E-03 | 1.77 |
| biological process | GO:0006402 | mRNA catabolic process | 6 | 6.34E-03 | 3.47 |
| biological process | GO:0034612 | response to tumor necrosis factor | 6 | 6.34E-03 | 3.47 |
| biological process | GO:0008630 | intrinsic apoptotic signaling pathway in response to DNA damage | 8 | 6.56E-03 | 2.82 |
| molecular function | GO:0001217 | DNA-binding transcription repressor activity | 4 | 6.57E-03 | 4.99 |
| biological process | GO:0010614 | negative regulation of cardiac muscle hypertrophy | 4 | 6.57E-03 | 4.99 |
| biological process | GO:0034145 | positive regulation of toll-like receptor 4 signaling pathway | 4 | 6.57E-03 | 4.99 |
| biological process | GO:0042129 | regulation of T cell proliferation | 4 | 6.57E-03 | 4.99 |
| molecular function | GO:0002151 | G-quadruplex RNA binding | 3 | 6.79E-03 | 6.95 |
| molecular function | GO:0005000 | vasopressin receptor activity | 3 | 6.79E-03 | 6.95 |
| biological process | GO:0014902 | myotube differentiation | 3 | 6.79E-03 | 6.95 |
| cellular component | GO:0016461 | unconventional myosin complex | 3 | 6.79E-03 | 6.95 |
| biological process | GO:0031394 | positive regulation of prostaglandin biosynthetic process | 3 | 6.79E-03 | 6.95 |
| cellular component | GO:0044327 | dendritic spine head | 3 | 6.79E-03 | 6.95 |
| biological process | GO:0061154 | endothelial tube morphogenesis | 3 | 6.79E-03 | 6.95 |
| biological process | GO:0072574 | hepatocyte proliferation | 3 | 6.79E-03 | 6.95 |
| biological process | GO:1904037 | positive regulation of epithelial cell apoptotic process | 3 | 6.79E-03 | 6.95 |
| biological process | GO:1904951 | positive regulation of establishment of protein localization | 3 | 6.79E-03 | 6.95 |
| cellular component | GO:0009897 | external side of plasma membrane | 35 | 7.04E-03 | 1.55 |
| biological process | GO:0034605 | cellular response to heat | 8 | 7.49E-03 | 2.76 |
| biological process | GO:0010575 | positive regulation of vascular endothelial growth factor production | 6 | 7.59E-03 | 3.35 |
| biological process | GO:0051602 | response to electrical stimulus | 6 | 7.59E-03 | 3.35 |

| | | | | | |
|---|---|---|---|---|---|
| biological process | GO:0019827 | stem cell population maintenance | 7 | 7.82E-03 | 2.99 |
| biological process | GO:0002042 | cell migration involved in sprouting angiogenesis | 5 | 7.86E-03 | 3.86 |
| biological process | GO:0007492 | endoderm development | 5 | 7.86E-03 | 3.86 |
| biological process | GO:0060218 | hematopoietic stem cell differentiation | 5 | 7.86E-03 | 3.86 |
| biological process | GO:0071539 | protein localization to centrosome | 5 | 7.86E-03 | 3.86 |
| biological process | GO:0090050 | positive regulation of cell migration involved in sprouting angiogenesis | 5 | 7.86E-03 | 3.86 |
| molecular function | GO:0004888 | transmembrane signaling receptor activity | 17 | 8.08E-03 | 1.90 |
| biological process | GO:0050728 | negative regulation of inflammatory response | 14 | 8.32E-03 | 2.04 |
| biological process | GO:0007266 | Rho protein signal transduction | 9 | 8.69E-03 | 2.52 |
| cellular component | GO:0035577 | azurophil granule membrane | 9 | 8.69E-03 | 2.52 |
| biological process | GO:0070102 | interleukin-6-mediated signaling pathway | 4 | 8.76E-03 | 4.63 |
| cellular component | GO:1990023 | mitotic spindle midzone | 4 | 8.76E-03 | 4.63 |
| biological process | GO:0001933 | negative regulation of protein phosphorylation | 11 | 8.96E-03 | 2.26 |
| biological process | GO:0002682 | regulation of immune system process | 7 | 9.04E-03 | 2.91 |
| biological process | GO:0008286 | insulin receptor signaling pathway | 10 | 9.43E-03 | 2.35 |
| biological process | GO:0034614 | cellular response to reactive oxygen species | 8 | 9.64E-03 | 2.65 |
| biological process | GO:0002862 | negative regulation of inflammatory response to antigenic stimulus | 5 | 9.66E-03 | 3.68 |
| biological process | GO:0070498 | interleukin-1-mediated signaling pathway | 5 | 9.66E-03 | 3.68 |
| biological process | GO:0002544 | chronic inflammatory response | 3 | 1.04E-02 | 6.08 |
| biological process | GO:0034141 | positive regulation of toll-like receptor 3 signaling pathway | 3 | 1.04E-02 | 6.08 |
| biological process | GO:0035457 | cellular response to interferon-alpha | 3 | 1.04E-02 | 6.08 |
| biological process | GO:0035767 | endothelial cell chemotaxis | 3 | 1.04E-02 | 6.08 |
| biological process | GO:0036289 | peptidyl-serine autophosphorylation | 3 | 1.04E-02 | 6.08 |
| biological process | GO:0042796 | snRNA transcription by RNA polymerase III | 3 | 1.04E-02 | 6.08 |
| biological process | GO:0045647 | negative regulation of erythrocyte differentiation | 3 | 1.04E-02 | 6.08 |
| biological process | GO:0046784 | viral mRNA export from host cell nucleus | 3 | 1.04E-02 | 6.08 |

| | | | | | |
|---|---|---|---|---|---|
| molecular function | GO:1990405 | protein antigen binding | 3 | 1.04E-02 | 6.08 |
| biological process | GO:0016266 | O-glycan processing | 7 | 1.04E-02 | 2.84 |
| biological process | GO:0071222 | cellular response to lipopolysaccharide | 19 | 1.05E-02 | 1.78 |
| biological process | GO:0001975 | response to amphetamine | 6 | 1.06E-02 | 3.14 |
| biological process | GO:0051085 | chaperone cofactor-dependent protein refolding | 6 | 1.06E-02 | 3.14 |
| biological process | GO:2001244 | positive regulation of intrinsic apoptotic signaling pathway | 6 | 1.06E-02 | 3.14 |
| molecular function | GO:0030544 | Hsp70 protein binding | 8 | 1.09E-02 | 2.59 |
| biological process | GO:0030838 | positive regulation of actin filament polymerization | 8 | 1.09E-02 | 2.59 |
| biological process | GO:0007064 | mitotic sister chromatid cohesion | 4 | 1.14E-02 | 4.32 |
| biological process | GO:0014002 | astrocyte development | 4 | 1.14E-02 | 4.32 |
| molecular function | GO:0030695 | GTPase regulator activity | 4 | 1.14E-02 | 4.32 |
| molecular function | GO:0045236 | CXCR chemokine receptor binding | 4 | 1.14E-02 | 4.32 |
| cellular component | GO:0001725 | stress fiber | 10 | 1.15E-02 | 2.28 |
| molecular function | GO:0019957 | C-C chemokine binding | 5 | 1.17E-02 | 3.52 |
| cellular component | GO:0005801 | cis-Golgi network | 9 | 1.20E-02 | 2.39 |
| biological process | GO:0032436 | positive regulation of proteasomal ubiquitin-dependent protein catabolic process | 11 | 1.28E-02 | 2.15 |
| cellular component | GO:0005879 | axonemal microtubule | 7 | 1.35E-02 | 2.70 |
| biological process | GO:0032689 | negative regulation of type II interferon production | 7 | 1.35E-02 | 2.70 |
| biological process | GO:0016925 | protein sumoylation | 8 | 1.37E-02 | 2.49 |
| biological process | GO:1902895 | positive regulation of miRNA transcription | 8 | 1.37E-02 | 2.49 |
| molecular function | GO:0042803 | protein homodimerization activity | 59 | 1.38E-02 | 1.34 |
| biological process | GO:0007099 | centriole replication | 5 | 1.41E-02 | 3.38 |
| biological process | GO:0051179 | localization | 5 | 1.41E-02 | 3.38 |
| biological process | GO:0050873 | brown fat cell differentiation | 6 | 1.43E-02 | 2.95 |
| molecular function | GO:0031625 | ubiquitin protein ligase binding | 29 | 1.44E-02 | 1.54 |
| biological process | GO:0002407 | dendritic cell chemotaxis | 4 | 1.44E-02 | 4.05 |

| | | | | | |
|---|---|---|---|---|---|
| molecular function | GO:0008373 | sialyltransferase activity | 4 | 1.44E-02 | 4.05 |
| cellular component | GO:0035102 | PRC1 complex | 4 | 1.44E-02 | 4.05 |
| biological process | GO:0051090 | regulation of DNA-binding transcription factor activity | 4 | 1.44E-02 | 4.05 |
| biological process | GO:0072520 | seminiferous tubule development | 4 | 1.44E-02 | 4.05 |
| biological process | GO:0090336 | positive regulation of brown fat cell differentiation | 4 | 1.44E-02 | 4.05 |
| biological process | GO:0071560 | cellular response to transforming growth factor beta stimulus | 9 | 1.47E-02 | 2.32 |
| biological process | GO:0001973 | G protein-coupled adenosine receptor signaling pathway | 3 | 1.49E-02 | 5.40 |
| biological process | GO:0006930 | substrate-dependent cell migration, cell extension | 3 | 1.49E-02 | 5.40 |
| cellular component | GO:0030915 | Smc5-Smc6 complex | 3 | 1.49E-02 | 5.40 |
| biological process | GO:0031509 | subtelomeric heterochromatin formation | 3 | 1.49E-02 | 5.40 |
| biological process | GO:0045217 | cell-cell junction maintenance | 3 | 1.49E-02 | 5.40 |
| biological process | GO:0050798 | activated T cell proliferation | 3 | 1.49E-02 | 5.40 |
| biological process | GO:0050805 | negative regulation of synaptic transmission | 3 | 1.49E-02 | 5.40 |
| biological process | GO:0030335 | positive regulation of cell migration | 23 | 1.54E-02 | 1.62 |
| biological process | GO:0006915 | apoptotic process | 47 | 1.57E-02 | 1.38 |
| cellular component | GO:0031410 | cytoplasmic vesicle | 26 | 1.64E-02 | 1.56 |
| biological process | GO:0017148 | negative regulation of translation | 11 | 1.64E-02 | 2.07 |
| biological process | GO:0043542 | endothelial cell migration | 6 | 1.65E-02 | 2.86 |
| cellular component | GO:0016605 | PML body | 13 | 1.67E-02 | 1.93 |
| molecular function | GO:0008157 | protein phosphatase 1 binding | 5 | 1.67E-02 | 3.24 |
| biological process | GO:0090023 | positive regulation of neutrophil chemotaxis | 5 | 1.67E-02 | 3.24 |
| cellular component | GO:1990904 | ribonucleoprotein complex | 18 | 1.67E-02 | 1.73 |
| cellular component | GO:0030864 | cortical actin cytoskeleton | 8 | 1.70E-02 | 2.40 |
| biological process | GO:0051301 | cell division | 32 | 1.70E-02 | 1.48 |
| biological process | GO:0007204 | positive regulation of cytosolic calcium ion concentration | 16 | 1.71E-02 | 1.79 |
| cellular component | GO:0005802 | trans-Golgi network | 19 | 1.73E-02 | 1.69 |

| | | | | | |
|---|---|---|---|---|---|
| biological process | GO:0006919 | activation of cysteine-type endopeptidase activity involved in apoptotic process | 9 | 1.78E-02 | 2.24 |
| biological process | GO:0008637 | apoptotic mitochondrial changes | 4 | 1.80E-02 | 3.81 |
| biological process | GO:0032206 | positive regulation of telomere maintenance | 4 | 1.80E-02 | 3.81 |
| biological process | GO:0045987 | positive regulation of smooth muscle contraction | 4 | 1.80E-02 | 3.81 |
| biological process | GO:0060271 | cilium assembly | 22 | 1.83E-02 | 1.61 |
| molecular function | GO:0008270 | zinc ion binding | 66 | 1.84E-02 | 1.30 |
| molecular function | GO:0005096 | GTPase activator activity | 26 | 1.86E-02 | 1.54 |
| biological process | GO:0031663 | lipopolysaccharide-mediated signaling pathway | 6 | 1.89E-02 | 2.78 |
| biological process | GO:0007015 | actin filament organization | 15 | 1.94E-02 | 1.80 |
| biological process | GO:0006493 | protein O-linked glycosylation | 9 | 1.95E-02 | 2.21 |
| cellular component | GO:0019898 | extrinsic component of membrane | 5 | 1.97E-02 | 3.12 |
| cellular component | GO:0000346 | transcription export complex | 3 | 2.03E-02 | 4.86 |
| biological process | GO:0002031 | G protein-coupled receptor internalization | 3 | 2.03E-02 | 4.86 |
| molecular function | GO:0004382 | GDP phosphatase activity | 3 | 2.03E-02 | 4.86 |
| biological process | GO:0006098 | pentose-phosphate shunt | 3 | 2.03E-02 | 4.86 |
| molecular function | GO:0008504 | monoamine transmembrane transporter activity | 3 | 2.03E-02 | 4.86 |
| biological process | GO:0009247 | glycolipid biosynthetic process | 3 | 2.03E-02 | 4.86 |
| biological process | GO:0010225 | response to UV-C | 3 | 2.03E-02 | 4.86 |
| biological process | GO:0032675 | regulation of interleukin-6 production | 3 | 2.03E-02 | 4.86 |
| biological process | GO:0032680 | regulation of tumor necrosis factor production | 3 | 2.03E-02 | 4.86 |
| biological process | GO:0033151 | V(D)J recombination | 3 | 2.03E-02 | 4.86 |
| molecular function | GO:0043422 | protein kinase B binding | 3 | 2.03E-02 | 4.86 |
| biological process | GO:0045063 | T-helper 1 cell differentiation | 3 | 2.03E-02 | 4.86 |
| biological process | GO:0045064 | T-helper 2 cell differentiation | 3 | 2.03E-02 | 4.86 |
| biological process | GO:0045670 | regulation of osteoclast differentiation | 3 | 2.03E-02 | 4.86 |
| biological process | GO:0046599 | regulation of centriole replication | 3 | 2.03E-02 | 4.86 |

| | | | | | |
|---|---|---|---|---|---|
| biological process | GO:0046825 | regulation of protein export from nucleus | 3 | 2.03E-02 | 4.86 |
| biological process | GO:0090084 | negative regulation of inclusion body assembly | 3 | 2.03E-02 | 4.86 |
| cellular component | GO:0120103 | centriolar subdistal appendage | 3 | 2.03E-02 | 4.86 |
| biological process | GO:0150077 | regulation of neuroinflammatory response | 3 | 2.03E-02 | 4.86 |
| biological process | GO:2000234 | positive regulation of rRNA processing | 3 | 2.03E-02 | 4.86 |
| cellular component | GO:1904813 | ficolin-1-rich granule lumen | 14 | 2.07E-02 | 1.83 |
| molecular function | GO:0038023 | signaling receptor activity | 19 | 2.12E-02 | 1.66 |
| biological process | GO:0000082 | G1/S transition of mitotic cell cycle | 9 | 2.14E-02 | 2.18 |
| cellular component | GO:0048471 | perinuclear region of cytoplasm | 57 | 2.15E-02 | 1.31 |
| biological process | GO:0120162 | positive regulation of cold-induced thermogenesis | 12 | 2.15E-02 | 1.93 |
| biological process | GO:0036297 | interstrand cross-link repair | 6 | 2.16E-02 | 2.70 |
| biological process | GO:0042127 | regulation of cell population proliferation | 15 | 2.19E-02 | 1.77 |
| biological process | GO:0010737 | protein kinase A signaling | 4 | 2.20E-02 | 3.60 |
| cellular component | GO:0031527 | filopodium membrane | 4 | 2.20E-02 | 3.60 |
| biological process | GO:0042692 | muscle cell differentiation | 4 | 2.20E-02 | 3.60 |
| biological process | GO:0061136 | regulation of proteasomal protein catabolic process | 4 | 2.20E-02 | 3.60 |
| biological process | GO:0071318 | cellular response to ATP | 4 | 2.20E-02 | 3.60 |
| biological process | GO:0007200 | phospholipase C-activating G protein-coupled receptor signaling pathway | 11 | 2.24E-02 | 1.98 |
| biological process | GO:0050727 | regulation of inflammatory response | 11 | 2.24E-02 | 1.98 |
| cellular component | GO:0000775 | chromosome, centromeric region | 8 | 2.30E-02 | 2.28 |
| biological process | GO:0038061 | non-canonical NF-kappaB signal transduction | 5 | 2.30E-02 | 3.00 |
| biological process | GO:0032760 | positive regulation of tumor necrosis factor production | 12 | 2.31E-02 | 1.91 |
| molecular function | GO:0004674 | protein serine/threonine kinase activity | 32 | 2.35E-02 | 1.44 |
| biological process | GO:0045893 | positive regulation of DNA-templated transcription | 55 | 2.40E-02 | 1.31 |
| molecular function | GO:0001784 | phosphotyrosine residue binding | 7 | 2.42E-02 | 2.41 |
| biological process | GO:0034446 | substrate adhesion-dependent cell spreading | 7 | 2.42E-02 | 2.41 |

| | | | | | |
|---|---|---|---|---|---|
| biological process | GO:0035914 | skeletal muscle cell differentiation | 7 | 2.42E-02 | 2.41 |
| biological process | GO:0031297 | replication fork processing | 6 | 2.45E-02 | 2.63 |
| biological process | GO:0071479 | cellular response to ionizing radiation | 6 | 2.45E-02 | 2.63 |
| molecular function | GO:0000979 | RNA polymerase II core promoter sequence-specific DNA binding | 4 | 2.65E-02 | 3.41 |
| biological process | GO:0006826 | iron ion transport | 4 | 2.65E-02 | 3.41 |
| biological process | GO:0019432 | triglyceride biosynthetic process | 4 | 2.65E-02 | 3.41 |
| biological process | GO:0046827 | positive regulation of protein export from nucleus | 4 | 2.65E-02 | 3.41 |
| biological process | GO:1904019 | epithelial cell apoptotic process | 4 | 2.65E-02 | 3.41 |
| biological process | GO:0019915 | lipid storage | 5 | 2.66E-02 | 2.89 |
| biological process | GO:0009101 | glycoprotein biosynthetic process | 3 | 2.66E-02 | 4.42 |
| biological process | GO:0019730 | antimicrobial humoral response | 3 | 2.66E-02 | 4.42 |
| biological process | GO:0042532 | negative regulation of tyrosine phosphorylation of STAT protein | 3 | 2.66E-02 | 4.42 |
| biological process | GO:0042795 | snRNA transcription by RNA polymerase II | 3 | 2.66E-02 | 4.42 |
| molecular function | GO:0045028 | G protein-coupled purinergic nucleotide receptor activity | 3 | 2.66E-02 | 4.42 |
| biological process | GO:0060020 | Bergmann glial cell differentiation | 3 | 2.66E-02 | 4.42 |
| biological process | GO:0060379 | cardiac muscle cell myoblast differentiation | 3 | 2.66E-02 | 4.42 |
| biological process | GO:0140588 | chromatin looping | 3 | 2.66E-02 | 4.42 |
| cellular component | GO:1905370 | serine-type endopeptidase complex | 3 | 2.66E-02 | 4.42 |
| biological process | GO:0031623 | receptor internalization | 7 | 2.68E-02 | 2.36 |
| biological process | GO:0043124 | negative regulation of canonical NF-kappaB signal transduction | 7 | 2.68E-02 | 2.36 |
| biological process | GO:0007165 | signal transduction | 87 | 2.70E-02 | 1.23 |
| cellular component | GO:0097431 | mitotic spindle pole | 6 | 2.76E-02 | 2.56 |
| biological process | GO:1900182 | positive regulation of protein localization to nucleus | 6 | 2.76E-02 | 2.56 |
| cellular component | GO:0016604 | nuclear body | 30 | 2.80E-02 | 1.44 |
| molecular function | GO:0005525 | GTP binding | 33 | 2.82E-02 | 1.42 |
| biological process | GO:0001764 | neuron migration | 12 | 2.82E-02 | 1.85 |

| | | | | | |
|---|---|---|---|---|---|
| molecular function | GO:0031267 | small GTPase binding | 26 | 2.88E-02 | 1.48 |
| biological process | GO:0007265 | Ras protein signal transduction | 10 | 2.91E-02 | 1.98 |
| biological process | GO:0006302 | double-strand break repair | 9 | 2.99E-02 | 2.05 |
| biological process | GO:0035556 | intracellular signal transduction | 33 | 3.00E-02 | 1.41 |
| biological process | GO:0032731 | positive regulation of interleukin-1 beta production | 8 | 3.03E-02 | 2.16 |
| cellular component | GO:0005912 | adherens junction | 18 | 3.09E-02 | 1.61 |
| cellular component | GO:0030496 | midbody | 18 | 3.09E-02 | 1.61 |
| molecular function | GO:0004896 | cytokine receptor activity | 6 | 3.10E-02 | 2.49 |
| biological process | GO:0033077 | T cell differentiation in thymus | 6 | 3.10E-02 | 2.49 |
| biological process | GO:0033627 | cell adhesion mediated by integrin | 6 | 3.10E-02 | 2.49 |
| molecular function | GO:0008093 | cytoskeletal anchor activity | 4 | 3.16E-02 | 3.24 |
| molecular function | GO:0010314 | phosphatidylinositol-5-phosphate binding | 4 | 3.16E-02 | 3.24 |
| biological process | GO:0032693 | negative regulation of interleukin-10 production | 4 | 3.16E-02 | 3.24 |
| biological process | GO:0043392 | negative regulation of DNA binding | 4 | 3.16E-02 | 3.24 |
| molecular function | GO:0003700 | DNA-binding transcription factor activity | 40 | 3.19E-02 | 1.35 |
| cellular component | GO:0070821 | tertiary granule membrane | 9 | 3.24E-02 | 2.03 |
| cellular component | GO:0031902 | late endosome membrane | 15 | 3.24E-02 | 1.69 |
| cellular component | GO:0005911 | cell-cell junction | 18 | 3.24E-02 | 1.60 |
| cellular component | GO:0005694 | chromosome | 20 | 3.27E-02 | 1.56 |
| biological process | GO:0045786 | negative regulation of cell cycle | 7 | 3.28E-02 | 2.27 |
| biological process | GO:0030168 | platelet activation | 8 | 3.30E-02 | 2.13 |
| cellular component | GO:0000347 | THO complex | 2 | 3.36E-02 | 6.48 |
| biological process | GO:0001992 | regulation of systemic arterial blood pressure by vasopressin | 2 | 3.36E-02 | 6.48 |
| biological process | GO:0002317 | plasma cell differentiation | 2 | 3.36E-02 | 6.48 |
| biological process | GO:0003176 | aortic valve development | 2 | 3.36E-02 | 6.48 |
| biological process | GO:0003209 | cardiac atrium morphogenesis | 2 | 3.36E-02 | 6.48 |

| | | | | | |
|---|---|---|---|---|---|
| molecular function | GO:0004366 | glycerol-3-phosphate O-acyltransferase activity | 2 | 3.36E-02 | 6.48 |
| biological process | GO:0006072 | glycerol-3-phosphate metabolic process | 2 | 3.36E-02 | 6.48 |
| biological process | GO:0006837 | serotonin transport | 2 | 3.36E-02 | 6.48 |
| molecular function | GO:0015651 | quaternary ammonium group transmembrane transporter activity | 2 | 3.36E-02 | 6.48 |
| molecular function | GO:0016019 | peptidoglycan immune receptor activity | 2 | 3.36E-02 | 6.48 |
| molecular function | GO:0019960 | C-X3-C chemokine binding | 2 | 3.36E-02 | 6.48 |
| biological process | GO:0030641 | regulation of cellular pH | 2 | 3.36E-02 | 6.48 |
| biological process | GO:0031579 | membrane raft organization | 2 | 3.36E-02 | 6.48 |
| biological process | GO:0031622 | positive regulation of fever generation | 2 | 3.36E-02 | 6.48 |
| cellular component | GO:0032010 | phagolysosome | 2 | 3.36E-02 | 6.48 |
| biological process | GO:0032792 | negative regulation of CREB transcription factor activity | 2 | 3.36E-02 | 6.48 |
| biological process | GO:0033299 | secretion of lysosomal enzymes | 2 | 3.36E-02 | 6.48 |
| biological process | GO:0034112 | positive regulation of homotypic cell-cell adhesion | 2 | 3.36E-02 | 6.48 |
| biological process | GO:0034184 | positive regulation of maintenance of mitotic sister chromatid cohesion | 2 | 3.36E-02 | 6.48 |
| molecular function | GO:0035662 | Toll-like receptor 4 binding | 2 | 3.36E-02 | 6.48 |
| molecular function | GO:0038132 | neuregulin binding | 2 | 3.36E-02 | 6.48 |
| biological process | GO:0038135 | ERBB2-ERBB4 signaling pathway | 2 | 3.36E-02 | 6.48 |
| biological process | GO:0042780 | tRNA 3'-end processing | 2 | 3.36E-02 | 6.48 |
| biological process | GO:0042976 | activation of Janus kinase activity | 2 | 3.36E-02 | 6.48 |
| molecular function | GO:0043125 | ErbB-3 class receptor binding | 2 | 3.36E-02 | 6.48 |
| molecular function | GO:0043262 | ADP phosphatase activity | 2 | 3.36E-02 | 6.48 |
| biological process | GO:0043270 | positive regulation of monoatomic ion transport | 2 | 3.36E-02 | 6.48 |
| biological process | GO:0045053 | protein retention in Golgi apparatus | 2 | 3.36E-02 | 6.48 |
| biological process | GO:0046601 | positive regulation of centriole replication | 2 | 3.36E-02 | 6.48 |
| biological process | GO:0050904 | diapedesis | 2 | 3.36E-02 | 6.48 |
| biological process | GO:0060087 | relaxation of vascular associated smooth muscle | 2 | 3.36E-02 | 6.48 |

| | | | | | |
|---|---|---|---|---|---|
| biological process | GO:0060354 | negative regulation of cell adhesion molecule production | 2 | 3.36E-02 | 6.48 |
| biological process | GO:0060591 | chondroblast differentiation | 2 | 3.36E-02 | 6.48 |
| biological process | GO:0060956 | endocardial cell differentiation | 2 | 3.36E-02 | 6.48 |
| biological process | GO:0070192 | chromosome organization involved in meiotic cell cycle | 2 | 3.36E-02 | 6.48 |
| biological process | GO:0070424 | regulation of nucleotide-binding oligomerization domain containing signaling pathway | 2 | 3.36E-02 | 6.48 |
| molecular function | GO:0070891 | lipoteichoic acid binding | 2 | 3.36E-02 | 6.48 |
| biological process | GO:0071376 | cellular response to corticotropin-releasing hormone stimulus | 2 | 3.36E-02 | 6.48 |
| biological process | GO:0072539 | T-helper 17 cell differentiation | 2 | 3.36E-02 | 6.48 |
| biological process | GO:0072584 | caveolin-mediated endocytosis | 2 | 3.36E-02 | 6.48 |
| cellular component | GO:0097728 | 9+0 motile cilium | 2 | 3.36E-02 | 6.48 |
| biological process | GO:0140206 | dipeptide import across plasma membrane | 2 | 3.36E-02 | 6.48 |
| molecular function | GO:0140346 | phosphatidylserine flippase activity | 2 | 3.36E-02 | 6.48 |
| molecular function | GO:0140444 | cytoskeleton-nuclear membrane anchor activity | 2 | 3.36E-02 | 6.48 |
| molecular function | GO:0140537 | transcription regulator activator activity | 2 | 3.36E-02 | 6.48 |
| biological process | GO:0140639 | positive regulation of pyroptosis | 2 | 3.36E-02 | 6.48 |
| biological process | GO:1902624 | positive regulation of neutrophil migration | 2 | 3.36E-02 | 6.48 |
| biological process | GO:1903724 | positive regulation of centriole elongation | 2 | 3.36E-02 | 6.48 |
| biological process | GO:1903895 | negative regulation of IRE1-mediated unfolded protein response | 2 | 3.36E-02 | 6.48 |
| biological process | GO:1904354 | negative regulation of telomere capping | 2 | 3.36E-02 | 6.48 |
| biological process | GO:1904358 | positive regulation of telomere maintenance via telomere lengthening | 2 | 3.36E-02 | 6.48 |
| biological process | GO:1990592 | protein K69-linked ufmylation | 2 | 3.36E-02 | 6.48 |
| biological process | GO:2001268 | negative regulation of cysteine-type endopeptidase activity involved in apoptotic signaling pathway | 2 | 3.36E-02 | 6.48 |
| biological process | GO:0002430 | complement receptor mediated signaling pathway | 3 | 3.39E-02 | 4.05 |
| biological process | GO:0002523 | leukocyte migration involved in inflammatory response | 3 | 3.39E-02 | 4.05 |

| | | | | | |
|---|---|---|---|---|---|
| molecular function | GO:0017166 | vinculin binding | 3 | 3.39E-02 | 4.05 |
| biological process | GO:0034453 | microtubule anchoring | 3 | 3.39E-02 | 4.05 |
| biological process | GO:0043117 | positive regulation of vascular permeability | 3 | 3.39E-02 | 4.05 |
| biological process | GO:0045616 | regulation of keratinocyte differentiation | 3 | 3.39E-02 | 4.05 |
| biological process | GO:0055008 | cardiac muscle tissue morphogenesis | 3 | 3.39E-02 | 4.05 |
| biological process | GO:0060396 | growth hormone receptor signaling pathway | 3 | 3.39E-02 | 4.05 |
| molecular function | GO:0070679 | inositol 1,4,5 trisphosphate binding | 3 | 3.39E-02 | 4.05 |
| biological process | GO:0090305 | nucleic acid phosphodiester bond hydrolysis | 3 | 3.39E-02 | 4.05 |
| cellular component | GO:0005815 | microtubule organizing center | 12 | 3.42E-02 | 1.80 |
| biological process | GO:0048488 | synaptic vesicle endocytosis | 6 | 3.46E-02 | 2.43 |
| biological process | GO:0007498 | mesoderm development | 5 | 3.48E-02 | 2.70 |
| biological process | GO:0034113 | heterotypic cell-cell adhesion | 5 | 3.48E-02 | 2.70 |
| biological process | GO:0043001 | Golgi to plasma membrane protein transport | 5 | 3.48E-02 | 2.70 |
| biological process | GO:0045773 | positive regulation of axon extension | 5 | 3.48E-02 | 2.70 |
| biological process | GO:0045907 | positive regulation of vasoconstriction | 5 | 3.48E-02 | 2.70 |
| biological process | GO:0048008 | platelet-derived growth factor receptor signaling pathway | 5 | 3.48E-02 | 2.70 |
| biological process | GO:1901223 | negative regulation of non-canonical NF-kappaB signal transduction | 5 | 3.48E-02 | 2.70 |
| molecular function | GO:0003779 | actin binding | 24 | 3.57E-02 | 1.48 |
| biological process | GO:0051260 | protein homooligomerization | 13 | 3.58E-02 | 1.74 |
| biological process | GO:0000281 | mitotic cytokinesis | 8 | 3.60E-02 | 2.09 |
| cellular component | GO:0030139 | endocytic vesicle | 8 | 3.60E-02 | 2.09 |
| biological process | GO:0032720 | negative regulation of tumor necrosis factor production | 8 | 3.60E-02 | 2.09 |
| molecular function | GO:0042277 | peptide binding | 7 | 3.60E-02 | 2.22 |
| cellular component | GO:0045111 | intermediate filament cytoskeleton | 7 | 3.60E-02 | 2.22 |
| cellular component | GO:0035861 | site of double-strand break | 10 | 3.61E-02 | 1.91 |
| biological process | GO:0000724 | double-strand break repair via homologous recombination | 11 | 3.64E-02 | 1.84 |

| | | | | | |
|---|---|---|---|---|---|
| molecular function | GO:0045296 | cadherin binding | 28 | 3.70E-02 | 1.43 |
| cellular component | GO:0005942 | phosphatidylinositol 3-kinase complex | 4 | 3.72E-02 | 3.09 |
| molecular function | GO:0030742 | GTP-dependent protein binding | 4 | 3.72E-02 | 3.09 |
| biological process | GO:0032204 | regulation of telomere maintenance | 4 | 3.72E-02 | 3.09 |
| biological process | GO:2000773 | negative regulation of cellular senescence | 4 | 3.72E-02 | 3.09 |
| molecular function | GO:0016887 | ATP hydrolysis activity | 34 | 3.76E-02 | 1.37 |
| molecular function | GO:0003725 | double-stranded RNA binding | 9 | 3.78E-02 | 1.97 |
| biological process | GO:0032733 | positive regulation of interleukin-10 production | 6 | 3.85E-02 | 2.37 |
| biological process | GO:0006897 | endocytosis | 17 | 3.88E-02 | 1.59 |
| molecular function | GO:0001664 | G protein-coupled receptor binding | 8 | 3.91E-02 | 2.06 |
| biological process | GO:0035176 | social behavior | 7 | 3.95E-02 | 2.18 |
| biological process | GO:0008104 | protein localization | 13 | 4.01E-02 | 1.71 |
| biological process | GO:0045727 | positive regulation of translation | 9 | 4.07E-02 | 1.95 |
| biological process | GO:0006606 | protein import into nucleus | 11 | 4.14E-02 | 1.80 |
| biological process | GO:0007096 | regulation of exit from mitosis | 3 | 4.21E-02 | 3.74 |
| biological process | GO:0010745 | negative regulation of macrophage derived foam cell differentiation | 3 | 4.21E-02 | 3.74 |
| biological process | GO:0021702 | cerebellar Purkinje cell differentiation | 3 | 4.21E-02 | 3.74 |
| biological process | GO:0035313 | wound healing, spreading of epidermal cells | 3 | 4.21E-02 | 3.74 |
| biological process | GO:0035589 | G protein-coupled purinergic nucleotide receptor signaling pathway | 3 | 4.21E-02 | 3.74 |
| cellular component | GO:0036038 | MKS complex | 3 | 4.21E-02 | 3.74 |
| biological process | GO:0038066 | p38MAPK cascade | 3 | 4.21E-02 | 3.74 |
| biological process | GO:0043589 | skin morphogenesis | 3 | 4.21E-02 | 3.74 |
| biological process | GO:0051412 | response to corticosterone | 3 | 4.21E-02 | 3.74 |
| biological process | GO:0051984 | positive regulation of chromosome segregation | 3 | 4.21E-02 | 3.74 |
| biological process | GO:0060213 | positive regulation of nuclear-transcribed mRNA poly(A) tail shortening | 3 | 4.21E-02 | 3.74 |
| biological process | GO:0060333 | type II interferon-mediated signaling pathway | 3 | 4.21E-02 | 3.74 |

| | | | | | |
|---|---|---|---|---|---|
| molecular function | GO:0061665 | SUMO ligase activity | 3 | 4.21E-02 | 3.74 |
| biological process | GO:1903238 | positive regulation of leukocyte tethering or rolling | 3 | 4.21E-02 | 3.74 |
| cellular component | GO:1990391 | DNA repair complex | 3 | 4.21E-02 | 3.74 |
| biological process | GO:0002250 | adaptive immune response | 18 | 4.26E-02 | 1.55 |
| biological process | GO:0007566 | embryo implantation | 6 | 4.26E-02 | 2.32 |
| biological process | GO:0006612 | protein targeting to membrane | 7 | 4.32E-02 | 2.14 |
| biological process | GO:0033209 | tumor necrosis factor-mediated signaling pathway | 7 | 4.32E-02 | 2.14 |
| cellular component | GO:0031258 | lamellipodium membrane | 4 | 4.33E-02 | 2.95 |
| biological process | GO:0036092 | phosphatidylinositol-3-phosphate biosynthetic process | 4 | 4.33E-02 | 2.95 |
| molecular function | GO:0030674 | protein-macromolecule adaptor activity | 12 | 4.34E-02 | 1.74 |
| biological process | GO:0006611 | protein export from nucleus | 5 | 4.45E-02 | 2.53 |
| biological process | GO:0030100 | regulation of endocytosis | 5 | 4.45E-02 | 2.53 |
| molecular function | GO:0071889 | 14-3-3 protein binding | 5 | 4.45E-02 | 2.53 |
| biological process | GO:0045766 | positive regulation of angiogenesis | 14 | 4.57E-02 | 1.64 |
| biological process | GO:0046777 | protein autophosphorylation | 15 | 4.63E-02 | 1.61 |
| molecular function | GO:0008017 | microtubule binding | 23 | 4.70E-02 | 1.45 |
| molecular function | GO:0003755 | peptidyl-prolyl cis-trans isomerase activity | 6 | 4.71E-02 | 2.26 |
| biological process | GO:0000018 | regulation of DNA recombination | 2 | 4.83E-02 | 5.40 |
| cellular component | GO:0000152 | nuclear ubiquitin ligase complex | 2 | 4.83E-02 | 5.40 |
| cellular component | GO:0000445 | THO complex part of transcription export complex | 2 | 4.83E-02 | 5.40 |
| biological process | GO:0002385 | mucosal immune response | 2 | 4.83E-02 | 5.40 |
| biological process | GO:0002438 | acute inflammatory response to antigenic stimulus | 2 | 4.83E-02 | 5.40 |
| biological process | GO:0002532 | production of molecular mediator involved in inflammatory response | 2 | 4.83E-02 | 5.40 |
| biological process | GO:0003344 | pericardium morphogenesis | 2 | 4.83E-02 | 5.40 |
| molecular function | GO:0004691 | cAMP-dependent protein kinase activity | 2 | 4.83E-02 | 5.40 |
| biological process | GO:0006297 | nucleotide-excision repair, DNA gap filling | 2 | 4.83E-02 | 5.40 |

| | | | | | |
|---|---|---|---|---|---|
| biological process | GO:0006398 | mRNA 3'-end processing by stem-loop binding and cleavage | 2 | 4.83E-02 | 5.40 |
| biological process | GO:0006596 | polyamine biosynthetic process | 2 | 4.83E-02 | 5.40 |
| biological process | GO:0006880 | intracellular sequestering of iron ion | 2 | 4.83E-02 | 5.40 |
| biological process | GO:0007171 | activation of transmembrane receptor protein tyrosine kinase activity | 2 | 4.83E-02 | 5.40 |
| biological process | GO:0008356 | asymmetric cell division | 2 | 4.83E-02 | 5.40 |
| molecular function | GO:0009378 | four-way junction helicase activity | 2 | 4.83E-02 | 5.40 |
| biological process | GO:0009437 | carnitine metabolic process | 2 | 4.83E-02 | 5.40 |
| biological process | GO:0009629 | response to gravity | 2 | 4.83E-02 | 5.40 |
| biological process | GO:0014842 | regulation of skeletal muscle satellite cell proliferation | 2 | 4.83E-02 | 5.40 |
| biological process | GO:0018344 | protein geranylgeranylation | 2 | 4.83E-02 | 5.40 |
| biological process | GO:0019563 | glycerol catabolic process | 2 | 4.83E-02 | 5.40 |
| biological process | GO:0021532 | neural tube patterning | 2 | 4.83E-02 | 5.40 |
| cellular component | GO:0030289 | protein phosphatase 4 complex | 2 | 4.83E-02 | 5.40 |
| biological process | GO:0030311 | poly-N-acetyllactosamine biosynthetic process | 2 | 4.83E-02 | 5.40 |
| cellular component | GO:0030892 | mitotic cohesin complex | 2 | 4.83E-02 | 5.40 |
| biological process | GO:0031442 | positive regulation of mRNA 3'-end processing | 2 | 4.83E-02 | 5.40 |
| biological process | GO:0031591 | wybutosine biosynthetic process | 2 | 4.83E-02 | 5.40 |
| molecular function | GO:0031802 | type 5 metabotropic glutamate receptor binding | 2 | 4.83E-02 | 5.40 |
| biological process | GO:0032814 | regulation of natural killer cell activation | 2 | 4.83E-02 | 5.40 |
| biological process | GO:0032911 | negative regulation of transforming growth factor beta1 production | 2 | 4.83E-02 | 5.40 |
| biological process | GO:0033081 | regulation of T cell differentiation in thymus | 2 | 4.83E-02 | 5.40 |
| biological process | GO:0034157 | positive regulation of toll-like receptor 7 signaling pathway | 2 | 4.83E-02 | 5.40 |
| biological process | GO:0034165 | positive regulation of toll-like receptor 9 signaling pathway | 2 | 4.83E-02 | 5.40 |
| biological process | GO:0035630 | bone mineralization involved in bone maturation | 2 | 4.83E-02 | 5.40 |
| biological process | GO:0042634 | regulation of hair cycle | 2 | 4.83E-02 | 5.40 |
| biological process | GO:0043367 | CD4-positive, alpha-beta T cell differentiation | 2 | 4.83E-02 | 5.40 |

| | | | | | |
|---|---|---|---|---|---|
| biological process | GO:0043372 | positive regulation of CD4-positive, alpha-beta T cell differentiation | 2 | 4.83E-02 | 5.40 |
| molecular function | GO:0043515 | kinetochore binding | 2 | 4.83E-02 | 5.40 |
| biological process | GO:0043535 | regulation of blood vessel endothelial cell migration | 2 | 4.83E-02 | 5.40 |
| biological process | GO:0045345 | positive regulation of MHC class I biosynthetic process | 2 | 4.83E-02 | 5.40 |
| biological process | GO:0045646 | regulation of erythrocyte differentiation | 2 | 4.83E-02 | 5.40 |
| biological process | GO:0045661 | regulation of myoblast differentiation | 2 | 4.83E-02 | 5.40 |
| biological process | GO:0051715 | cytolysis in another organism | 2 | 4.83E-02 | 5.40 |
| molecular function | GO:0055131 | C3HC4-type RING finger domain binding | 2 | 4.83E-02 | 5.40 |
| biological process | GO:0061002 | negative regulation of dendritic spine morphogenesis | 2 | 4.83E-02 | 5.40 |
| biological process | GO:0061038 | uterus morphogenesis | 2 | 4.83E-02 | 5.40 |
| molecular function | GO:0070087 | chromo shadow domain binding | 2 | 4.83E-02 | 5.40 |
| cellular component | GO:0070533 | BRCA1-C complex | 2 | 4.83E-02 | 5.40 |
| cellular component | GO:0070938 | contractile ring | 2 | 4.83E-02 | 5.40 |
| biological process | GO:0071569 | protein ufmylation | 2 | 4.83E-02 | 5.40 |
| biological process | GO:0072655 | establishment of protein localization to mitochondrion | 2 | 4.83E-02 | 5.40 |
| molecular function | GO:0090555 | phosphatidylethanolamine flippase activity | 2 | 4.83E-02 | 5.40 |
| biological process | GO:0097503 | sialylation | 2 | 4.83E-02 | 5.40 |
| molecular function | GO:0106411 | XMP 5'-nucleosidase activity | 2 | 4.83E-02 | 5.40 |
| cellular component | GO:0120099 | procentriole replication complex | 2 | 4.83E-02 | 5.40 |
| biological process | GO:0150011 | regulation of neuron projection arborization | 2 | 4.83E-02 | 5.40 |
| biological process | GO:1902083 | negative regulation of peptidyl-cysteine S-nitrosylation | 2 | 4.83E-02 | 5.40 |
| biological process | GO:1990962 | xenobiotic transport across blood-brain barrier | 2 | 4.83E-02 | 5.40 |
| biological process | GO:0001525 | angiogenesis | 22 | 4.97E-02 | 1.46 |
| biological process | GO:0071356 | cellular response to tumor necrosis factor | 13 | 4.98E-02 | 1.66 |
| biological process | GO:0001556 | oocyte maturation | 4 | 4.99E-02 | 2.82 |
| biological process | GO:0002223 | stimulatory C-type lectin receptor signaling pathway | 4 | 4.99E-02 | 2.82 |

| | | | | | |
|---|---|---|---|---|---|
| molecular function | GO:0016493 | C-C chemokine receptor activity | 4 | 4.99E-02 | 2.82 |
| biological process | GO:0018345 | protein palmitoylation | 4 | 4.99E-02 | 2.82 |
| biological process | GO:0019934 | cGMP-mediated signaling | 4 | 4.99E-02 | 2.82 |
| molecular function | GO:0051019 | mitogen-activated protein kinase binding | 4 | 4.99E-02 | 2.82 |
| biological process | GO:2001243 | negative regulation of intrinsic apoptotic signaling pathway | 4 | 4.99E-02 | 2.82 |
| biological process | GO:0035025 | positive regulation of Rho protein signal transduction | 5 | 4.99E-02 | 2.46 |
| biological process | GO:0071276 | cellular response to cadmium ion | 5 | 4.99E-02 | 2.46 |
