# Supplementary material for: Transcriptomic analysis reveals the key role of inflammatory and immune signaling in the anti-perimenopausal depression effects of Bushen Shugan Huayu decoction
Source: Front Psychiatry. 2025 Sep 26;16:1629900. doi: 10.3389/fpsyt.2025.1629900 (PMC12512047; doi:10.3389/fpsyt.2025.1629900)
Supplement: Supplementary file 4 [file Table4.pdf]

Table S4 KEGG enrichment analysis (*p*-value < 0.05). 41 KEGG terms are identified using the filter *p* < 0.05. The term, the number of DEGs, *p*-value, and the enrichment score (ES) are described in the table.

Table S4 KEGG enrichment analysis (*p*-value < 0.05)

ID	Term	Number	*p*-value	ES
hsa00030	Pentose phosphate pathway	5	0.04	2.57
hsa01522	Endocrine resistance	11	0.04	1.79
hsa03450	Non-homologous end-joining	3	0.05	3.56
hsa04010	MAPK signaling pathway	30	0.01	1.54
hsa04061	Viral protein interaction with cytokine and cytokine receptor	12	0.02	1.89
hsa04062	Chemokine signaling pathway	27	0.00	2.19
hsa04072	Phospholipase D signaling pathway	16	0.03	1.68
hsa04210	Apoptosis	16	0.01	1.83
hsa04216	Ferroptosis	8	0.00	3.01
hsa04217	Necroptosis	17	0.03	1.65
hsa04380	Osteoclast differentiation	23	0.00	2.69
hsa04520	Adherens junction	11	0.04	1.82
hsa04540	Gap junction	11	0.03	1.93
hsa04611	Platelet activation	17	0.00	2.11
hsa04621	NOD-like receptor signaling pathway	20	0.02	1.67
hsa04625	C-type lectin receptor signaling pathway	13	0.02	1.93
hsa04657	IL-17 signaling pathway	15	0.00	2.46
hsa04659	Th17 cell differentiation	12	0.04	1.75
hsa04662	B cell receptor signaling pathway	11	0.01	2.09
hsa04668	TNF signaling pathway	19	0.00	2.57

hsa04670	Leukocyte transendothelial migration	15	0.01	2.01
hsa04915	Estrogen signaling pathway	16	0.01	1.81
hsa04926	Relaxin signaling pathway	15	0.02	1.79
hsa04928	Parathyroid hormone synthesis, secretion and action	14	0.01	2.04
hsa04935	Growth hormone synthesis, secretion and action	14	0.02	1.80
hsa05120	Epithelial cell signaling in Helicobacter pylori infection	10	0.01	2.20
hsa05132	Salmonella infection	25	0.02	1.55
hsa05134	Legionellosis	8	0.03	2.20
hsa05135	Yersinia infection	16	0.01	1.81
hsa05140	Leishmaniasis	11	0.01	2.32
hsa05142	Chagas disease	13	0.01	1.99
hsa05146	Amoebiasis	13	0.01	1.99
hsa05161	Hepatitis B	17	0.03	1.63
hsa05163	Human cytomegalovirus infection	25	0.01	1.73
hsa05167	Kaposi sarcoma-associated herpesvirus infection	22	0.01	1.75
hsa05168	Herpes simplex virus 1 infection	57	0.00	1.72
hsa05200	Pathways in cancer	50	0.00	1.46
hsa05202	Transcriptional misregulation in cancer	20	0.02	1.61
hsa05205	Proteoglycans in cancer	20	0.04	1.53
hsa05235	PD-L1 expression and PD-1 checkpoint pathway in cancer	11	0.03	1.91
hsa05417	Lipid and atherosclerosis	24	0.01	1.73
