# Supplementary material for: Transcriptomic analysis reveals the key role of inflammatory and immune signaling in the anti-perimenopausal depression effects of Bushen Shugan Huayu decoction
Source: Front Psychiatry. 2025 Sep 26;16:1629900. doi: 10.3389/fpsyt.2025.1629900 (PMC12512047; doi:10.3389/fpsyt.2025.1629900)
Supplement: Supplementary file 5 [file Table5.pdf]

Table S5 key pathway GSEA analysis. GSEA analysis of the top 30 GO terms and KEGG pathways ($p < 0.05$) associated with inflammation and immunity. The term, the enrichment score (ES), normalized enrichment score (NES), *p*-value, and the FDR of GO/KEGG terms are described in the table.

Table S5 key pathway GSEA analysis

Term	ES	NES	*p*-value	FDR
integrated stress response signaling(GO:0140467)	-0.35	-1.212818342	0.19	0.72
positive regulation of intracellular protein transport(GO:0090316)	0.42	1.24623067	0.18	0.91
positive regulation of inflammatory response(GO:0050729)	-0.30	-1.47704669	0.01	0.51
inflammatory response(GO:0006954)	-0.24	-1.327289983	0.00	0.66
transcription factor AP-1 complex(GO:0035976)	-0.78	-1.635162643	0.04	0.67
cytosol(GO:0005829)	0.22	1.049722891	0.13	0.88
protein binding(GO:0005515)	0.17	0.817327795	1.00	1.00
Chemokine signaling pathway(hsa04062)	-0.26	-1.367545014	0.02	0.37
TNF signaling pathway(hsa04668)	-0.21	-0.993954476	0.48	1.00
IL-17 signaling pathway(hsa04657)	-0.36	-1.654785153	0.00	0.15
MAPK signaling pathway(hsa04010)	-0.19	-1.018441435	0.40	0.95
Estrogen signaling pathway(hsa04915)	-0.22	-1.073738005	0.27	0.76
Transcriptional misregulation in cancer(hsa05202)	-0.15	-0.77711147	0.98	1.00
PD-L1 expression and PD-1 checkpoint pathway in cancer(hsa05235)	0.17	0.661719751	0.99	1.00
Proteoglycans in cancer(hsa05205)	-0.19	-0.972033028	0.55	1.00
Th17 cell differentiation(hsa04659)	-0.26	-1.212658456	0.12	0.60
Pathways in cancer(hsa05200)	0.15	0.708213795	1.00	1.00
