# Supplementary material for: Transcriptomic analysis reveals the key role of inflammatory and immune signaling in the anti-perimenopausal depression effects of Bushen Shugan Huayu decoction
Source: Front Psychiatry. 2025 Sep 26;16:1629900. doi: 10.3389/fpsyt.2025.1629900 (PMC12512047; doi:10.3389/fpsyt.2025.1629900)
Supplement: Supplementary file 6 [file Table6.pdf]

Table S6 Coregenes of the top 5 GO/KEGG pathways. GO and KEGG pathways were screened based on p-values, with the top five pathways selected for PPI network analysis. From the gene interaction results, 10 core genes were identified within each path.

Table S6 Coregenes of top 5 GO/KEGG pathways

Categories	Term	Gene
GO-MF	protein binding	FOS, MAPK3, PRKACB, JUN, UBC, JAK2, CXCL1, NFKBIA, PRKACA, PRKACB
GO-CC	nucleus	MAPK3, FOS, JUND, JUN, UBC, H3-3B, BRCA1, ATM, H2BC21, H2AC18
GO-MF	RNA polymerase II cis-regulatory region sequence-specific DNA binding	CEBPE, EGR1, EGR3, JUND, EGR2, JUN, SPI1, FOS, FOSL1, RARA
GO-CC	cytosol	PRKACA, MAPK3, PRKACB, UBC, CCNA2, SOS1, NFKBIA, CCNA2, PIK3R1, IL1B
GO-CC	centriolar satellite	CCDC66, CSPP1, NEK1, CEP290, OFD1, PCM1, SSX2IP, CCDC14, PIBF1, CEP126
KEGG	Osteoclast differentiation	JUNB, FOSL1, FOS, JUND, JUNB, IL1B, NFKBIA, SOSCS3, MAPK3, TNFRSF1A
KEGG	Herpes simplex virus 1 infection	SOCS3, IFNGR2, JAK2, IL1B, TNFRSF1A, NFKBIA, BIRC3, FASLG, BID, IFIH1
KEGG	Chemokine signaling pathway	CXCL8, CXCR4, ARRB2, CCR1, CXCR1, CXCL3, CXCL1, CXCL11, PIK3R1, MAPK3
KEGG	TNF signaling pathway	JUN, IL1B, MMP9, PTGS2, SOCS3, CEBPB, FOS, NFKBIA, TNFRSF1A, TNFRSF1B
KEGG	IL-17 signaling pathway	FOS, JUN, FOSL1, MAPK3, JUND, CXCLB, CXCL1, MMP9, MAPK3, CEBPB
